# Nitrogen dynamics 35 years after stand‐replacing fire: Aboveground pools grow as surface soil pools remain unchanged

**DOI:** 10.1002/ecy.70546

**Published:** 2026-10-06

**Authors:** Timon T. Keller, Cory C. Cleveland, Robert E. Heumann, Arielle C. Link, Lucille T. McGuire, Monica G. Turner

**Affiliations:** ^1^ Department of Biology University of Wisconsin‐Madison Madison Wisconsin USA; ^2^ Department of Ecosystem and Conservation Sciences, W.A. Franke College of Forestry and Conservation University of Montana Missoula Montana USA; ^3^ Present address: Department of Geography and the Environment University of Alabama Tuscaloosa Alabama USA

**Keywords:** climate change, disturbance, ecosystem function, ecosystem processes, forest ecology, Greater Yellowstone, nitrogen cycling, wildfire

## Abstract

Natural disturbances induce variable recovery pathways, and long‐term studies are key to understanding how that variability affects ecosystem function over time and across space. In subalpine forests, high‐severity wildfires produce high variability in postfire tree density, but how this variability relates to nitrogen (N) cycling remains unclear. We resampled lodgepole pine (*Pinus contorta* var. *latifolia*) stands 35 years after stand‐replacing fire and asked (1) how N concentrations, pools, and mineralization changed with increasing lodgepole pine biomass from 15 to 35 years postfire; (2) how N concentrations, pools, and rates of change varied across the gradient of early postfire stand density; and (3) whether among‐stand structure and function were converging over time. Vegetation, forest floor litter, downed coarse wood, surface mineral soil (0–15 cm), and resin‐sorbed N were measured in fourteen 0.25‐ha plots in Yellowstone National Park (Wyoming, USA). From 25 to 35 years postfire, average total aboveground N pools increased from 252 to 405 kg N ha^−1^. Over the same 10‐year period, soil N pools averaged 986 kg N ha^−1^ and did not change, resin‐sorbed soil N remained low, and the only common symbiotic N‐fixing plant in the plots (silvery lupine, *Lupinus argenteus*) remained sparse. Aboveground lodgepole pine biomass increased at similar rates from 15 to 25 and 25 to 35 years postfire (2.5 and 2.2 Mg ha^−1^ year^−1^, respectively) along with N pools (4.6 and 3.8 kg N ha^−1^ year^1^) and forest floor litter N (1.2 and 2.2 kg N ha^−1^ year^1^). Among stands 35 years postfire, total aboveground N varied threefold (212–638 kg N ha^−1^) and was positively correlated with lodgepole pine aboveground net primary productivity and biomass but unrelated to stem density. Soil N pools ranged from 570 to 1668 kg N ha^−1^ and were negatively correlated with lodgepole pine stem density but unrelated to tree productivity or biomass. Among‐stand variation in stem density remained high but declined over time while N pools became less variable even as pools increased and variability in tree productivity increased from 25 to 35 years postfire. Rapid tree growth and biomass accumulation strongly regulate the N cycle in 35‐year‐old lodgepole pine stands, but sources of recovered N remain unresolved.

## INTRODUCTION

Natural disturbances induce variable recovery pathways in many ecosystems (Fraterrigo & Rusak, 2008; White, 1979), and long‐term studies are key to understanding how this variation affects the function of recovering ecosystems over time and across space (Gaiser et al., 2020; Turner et al., 2003). For example, recent long‐term studies revealed that disturbance legacies explain variation in productivity in giant kelp forests (Beckley et al., 2026) and vegetation recovery following landslides (Hotta et al., 2024) and volcanic eruptions (Denton et al., 2025). In subalpine and boreal forest landscapes shaped by high‐severity fire, early seral forests are becoming more prominent as annual area burned increases with warmer and drier climate (Abatzoglou & Williams, 2016; Parks & Abatzoglou, 2020; Wang et al., 2025). Yet, how forest ecosystem processes change through time and vary across space following stand‐replacing fire is not well understood (McDowell et al., 2020; Swanson et al., 2011; Waldron et al., 2023). Nitrogen (N) cycling is a critical ecosystem process that has been well studied in mature forests (Johnson & Turner, 2014; Lupi et al., 2013), and how fire initially affects N pools and disrupts cycles of N is also well understood (Grier, 1975; Grogan et al., 2000; Gustine et al., 2022; Raison, 1979; Tierney et al., 2019; Vitousek et al., 1979). However, few studies have tracked the trajectory of N pools and N cycling dynamics over time in forests adapted to stand‐replacing fire, such as subalpine and boreal forests (Ibáñez et al., 2022; Smithwick et al., 2005). Long‐term studies of N cycling after stand‐replacing fire offer rare opportunities to understand how forest ecosystems acquire, cycle, and retain critical nutrients as they recover from disturbance.

Stand‐replacing fires alter N pools and fluxes in subalpine forests in several ways. Aboveground N is lost via combustion of fine fuels such as live foliage and forest floor organic material (Johnson & Turner, 2014; Smithwick et al., 2009), and these losses can be substantial. For example, N pools in aboveground live tree and forest floor organic material (pools vulnerable to loss via combustion) averaged 87 and 153 kg N ha^−1^, respectively, across a 300‐year lodgepole pine (*Pinus contorta* var. *latifolia*) chronosequence in Yellowstone National Park (Smithwick et al., 2009), where stand‐replacing fires occurred at 100‐ to 300‐year intervals throughout the Holocene (Millspaugh et al., 2000). Losses from woody biomass and mineral soil N pools are generally low (Donato et al., 2006; Maynard et al., 2014; Tinker & Knight, 2000; Turner et al., 1999). However, stand‐replacing fire triggers pulses of available soil ammonium (NH_4_^+^) and nitrate (NO_3_^−^) (Christensen, 1973; Grogan et al., 2000) that may be vulnerable to loss via leaching (Rhoades et al., 2019; Vitousek et al., 1979) until recovering microbial biomass and rapidly growing young trees become strong N sinks (Chapman et al., 2006). Accumulating needle litter and downed coarse wood, consisting largely of fallen fire‐killed snags, become additional N sinks during decomposition (Fahey, 1983). As trees grow, live biomass N pools recover, and rates of biomass N recovery are tightly linked to stand productivity (Turner et al., 2009, 2019).

Variation in productivity among postfire stand development trajectories may shape N recovery over time and space. Leaf area index (LAI) and thus rates of aboveground net primary productivity (ANPP) in subalpine and boreal forests peak 40–70 years after stand establishment (Goulden et al., 2011; Kashian et al., 2013; Kashian, Turner, & Romme, 2005; Ryan et al., 1997). Peak rates of ANPP are greater and occur sooner after fire in dense, productive stands relative to sparse, less productive stands (Kashian et al., 2006; Turner, 2010). Nitrogen dynamics are often presumed to shape these patterns of subalpine forest productivity due to periodic N losses via stand‐replacing fire and relatively low inputs via N fixation (Fahey et al., 1985; Hogberg et al., 2017). However, rates of N pool recovery in postfire forests can exceed known N inputs, and N does not appear to constrain productivity in young postfire stands (Hupperts et al., 2025; Turner et al., 2019). Instead, stand productivity and biomass accumulation, which are closely linked to postfire tree establishment density in the first decades postfire (Turner et al., 2016), may affect N dynamics over time and among stands. Over time, rapid tree biomass accumulation and accompanying live biomass N recovery (Bond‐Lamberty et al., 2006; Larjavaara et al., 2017; Turner et al., 2019) should coincide with declining mineral soil N pools if N inputs (e.g., fixation and deposition) are insufficient to meet tree N demand (Lovett et al., 2018). Among stands, live N pools should recover faster, and soil N pools decline more strongly, in dense, productive stands unless inputs via biological N fixation offset these patterns (Gou et al., 2023; Vitousek et al., 2013). Yet, among‐stand variation in N pools could be reduced by convergence over time as dense stands self‐thin and sparse stands infill (Kashian, Turner, Romme, & Lorimer, 2005; Turner et al., 2016).

Long‐term, site‐based research studies can offer valuable insights into the recovery of N pools over time and variation among postfire stand development trajectories. Prior studies in Yellowstone National Park quantified N pools, concentrations, and availability 15 and 25 years after the 1988 Yellowstone Fires (Turner et al., 2009, 2019). Twenty‐five years postfire, the live lodgepole pine biomass N pool was nearly 80% of mean live lodgepole pine biomass N pools across a 300‐year chronosequence (Smithwick et al., 2009), and trees sustained high rates of ANPP (Turner et al., 2009, 2019). Soil N availability declined from 15 to 25 years postfire, but aboveground live biomass N pools continued to increase without commensurate declines in soil N pools (Turner et al., 2019) or increases in known N inputs (Heumann et al., 2025). Here, we extend this long‐term record of N concentrations, pools, and availability to 35 years after the fires. Specifically, we asked:

1. *Change over time*: How have N concentrations, N pools, and N mineralization changed as lodgepole pine biomass increased from 15 to 35 years postfire?
  As trees continue to grow, we expected demand‐driven increases in aboveground N pools and parallel declines in belowground pools and N availability.
2. *Spatial variation (among‐stand development trajectories)*: How do N concentrations, N pools, and rates of change vary among stands at 35 years postfire, and have these relationships changed over time? We expected aboveground N pools to increase with stem density, and by extension with tree biomass and productivity. Denser, more productive stands should result in stronger declines of soil N pools.
3. *Time × space*: Are measures of stand structure (e.g., stem density) or stand function (e.g., ANPP) showing evidence of convergence from 15 to 35 years postfire? We expected that among‐stand variation in indicators of ecosystem function would decrease by 35 years postfire, whereas among‐stand variation in stand structure would not have changed. Prior studies suggest that stands converge in function within a century and in structure by 200 years postfire as dense stands self‐thin while sparse stands infill (Kashian et al., 2013; Turner et al., 2016).

## METHODS

### Study area

Yellowstone National Park (“Yellowstone” from here on) in northwestern Wyoming, USA, encompasses ~9000 km^2^ centered on a high‐elevation (~2100–2700 m above sea level) plateau. Soils are predominantly Inceptisols of volcanic origin that include rhyolite and andesite (Rodman et al., 1996). Thirty‐year climate normals (1991–2020) at the centrally located Old Faithful weather station (44.456667° N, 110.849444° W) show an average January temperature of −9.6°C and an average July temperature of 14.2°C (NOAA, 2022). Precipitation occurs mainly in the winter as snowfall, and N deposition is 2–3 kg N ha^−1^ year^−1^ (Hoffman et al., 2019; National Atmospheric Deposition Program, 2025).

Climate has limited fire occurrence in Yellowstone throughout the Holocene, with large fires historically occurring every 100–300 years during hot, dry conditions (Millspaugh et al., 2000; Whitlock, 1993). This fire regime favors lodgepole pine, which dominates forests of the Yellowstone plateau (Romme, 1982). Other tree species, including quaking aspen (*Populus tremuloides*), Douglas‐fir (*Pseudotsuga menziesii*), subalpine fir (*Abies lasiocarpa*), Engelmann spruce (*Picea engelmannii*), and whitebark pine (*Pinus albicaulis*), can be locally present. Large wildfires affected nearly 36% of Yellowstone National Park in 1988, and postfire forest recovery has been tracked since 1989 (Turner et al., 1994, 1997, 1999, 2004, 2016).

### Field sampling

During summers 2022 and 2023, we measured annual resin‐sorbed N and resampled tree density, size, and biomass; litter and downed coarse wood biomass; and surface soil total N (to 15 cm) to estimate ecosystem N pools in 14 long‐term study plots that span the range of tree establishment density after the 1988 fires (Figure 1). We followed the same protocols used in the previous studies (Nelson et al., 2016; Turner et al., 2009, 2019), which are briefly summarized here. Plots are 50 m × 50 m and oriented due north on flat terrain (i.e., minimal slope and aspect). Within three 50 m × 2 m belt transects per plot, we counted all trees by size class (seedlings <15 cm in height, saplings 15 cm to 1.4 m height (dbh), and trees greater than dbh). Along each transect, we recorded dbh and basal diameter for eight to nine trees (*n* = 25 trees plot^−1^). Coarse wood biomass was sampled by recording decay class and diameter of downed wood ≥7.5 cm in diameter along three 30‐m Brown's transects oriented at 0°, 120°, and 240° azimuths to avoid sampling bias from non‐randomly oriented logs (Brown, 1974, 1982; Harmon et al., 2004). Percent cover of understory vascular vegetation, mosses, and lichen was recorded by species along with percent cover of uncharred litter, charred litter, bare mineral soil, rock, and wood in twenty‐five 0.25‐m^2^ quadrats along the transects. In postfire forests of Yellowstone, herbaceous plant cover is relatively sparse and averages ~30% (Kiel, Braziunas, & Turner, 2023; Romme et al., 2016).

**FIGURE 1:**
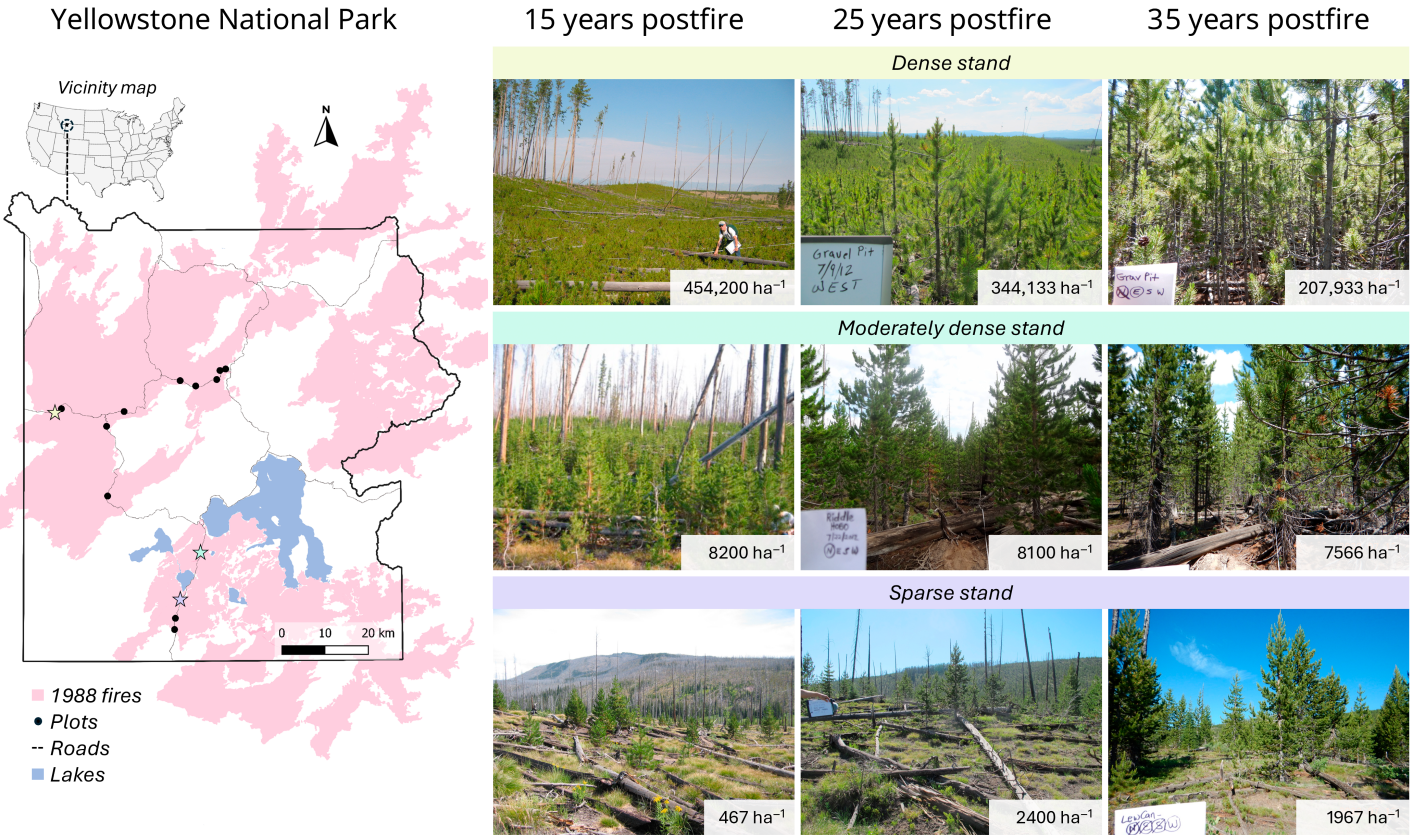
Locations of the 14 long‐term study plots in Yellowstone National Park (left) and photos of three long‐term study plots that illustrate the range of postfire stem densities at 15, 25, and 35 years after the 1988 Yellowstone fires. Each row shows the same plot at the three times postfire, and stars on the map highlight the locations of the three illustrative plots. All plots are located in areas that burned at high severity in the 1988 Yellowstone fires. All photo credit: Monica G. Turner.

For sampling litter N, soil N, and N mineralization, we divided plots into four 25 m × 25 m quadrants. In 2022, litter and soil samples were collected 3 m from quadrant centers in three sub‐cardinal directions (northeast, southeast, and southwest). Litter layers in aggrading postfire lodgepole pine forests are shallow, with an average depth of <2 cm 24 years postfire in the GYE (Nelson et al., 2016). All nonwoody litter was collected in 27.5 cm × 27.5 cm quadrants, dried at 60°C in the lab, and the mass recorded. Subsamples were then ground to a fine powder and analyzed for C and N content. Adjacent to litter sampling points, soil samples were obtained to 15 cm depth (2‐cm diameter) and composited by quadrant. In the lab, samples were sieved to 2 mm, dried at 105°C, ground to a fine powder, and analyzed for total C and N using a ThermoFisher FlashSmart Elemental Analyzer (no. 11206105). We also measured soil texture (percent sand, silt, and clay) using the hydrometer method (Bouyoucos, 1962). In 2023, soil bulk density was sampled in each quadrant to 15 cm depth using a 5‐cm‐diameter corer and averaged by plot (Jurgensen et al., 2017). To index plant‐available N, four free resin bags, constructed from undyed nylon stockings containing 20 g of mixed‐bed ion‐exchange resin (J. T. Baker no. JT4631‐1; Phillipsburg, NJ, USA), were deployed in each quadrant in July 2022 (*n* = 16 per plot). Free resin bags were used as an index of soil N availability because they are effective in remote settings (Binkley et al., 1986, 1992) and correlate well with more labor‐intensive measurements of net N mineralization (Binkley et al., 1986; Binkley & Matson, 1983). Prior to incubation, resin bags were cleaned by extracting in 2 M KCl and rinsing with deionized water. In the field, resin bags were positioned 2 m from the quadrant center in each cardinal direction (north, east, south, west) and gently inserted to a depth of ~5 cm. Resin bags were incubated in situ for a full year to align with the available inorganic N that trees could have accessed during the year before we sampled foliar [N]. In July 2023, resin bags were retrieved, gently cleaned of debris, and refrigerated until they were extracted in 2 M KCl and analyzed for ammonium and nitrate on a FIAlyzer‐1000 flow injection analyzer at the Soil and Forage Analysis Laboratory at the University of Wisconsin‐Madison.

Foliar N samples were collected in July 2023 to correspond with resin bag retrievals. For lodgepole pine, we harvested one branch each from a low, middle, and high position of eight lodgepole pines per plot. All needles were stripped from each branch, composited by tree, and air‐dried in the field. Needles were further dried at 60°C in the laboratory and analyzed for foliar N and C following the methods described above for litter. For understory vegetation, we identified the five most abundant vascular plant species in each plot in 2022 and harvested all aboveground biomass (most of which was foliage) from at least five individuals of each species in 2023. Samples were composited by species and plot, air‐dried in the field, returned to the laboratory, and prepared and processed for C and N analysis as described above for litter samples. Collectively, the top five species accounted for 72%–100% of the total live biotic cover in each plot (Appendix S1: Table S1).

### Calculations for stem density, aboveground biomass, and ecosystem N pools

To estimate N pools (in kilograms of nitrogen per hectare), we first calculated the biomass (in megagrams per hectare) of individual pools and multiplied by N concentrations as in Turner et al. (2009, 2019, 2025). Total ecosystem N pools were estimated by summing individual pools in each plot. Stand density (trees and saplings, in stems per hectare) was calculated per transect then averaged by plot.

Aboveground lodgepole pine biomass and ANPP were estimated from stand density and tree measurements using locally derived allometric equations (Copenhaver & Tinker, 2014) or standard equations (Pearson et al., 1987) for larger trees (>14 cm dbh, the largest trees included in locally derived equations). Because some measured trees exceeded the maximum size of trees used to develop the local allometric equations, we tested three methods for computing ANPP for the largest trees in our dataset (Appendix S2). There were minimal differences among methods (Appendix S2: Figure S1), and we proceeded by “trimming” the size of the largest trees in our dataset to the maximum tree size in Copenhaver and Tinker's (2014) dataset. We estimated aboveground biomass of understory vegetation using species‐level allometric equations that we developed previously (Turner et al., 2004, 2016, 2025). Downed coarse wood biomass was calculated from wood volume and wood density of each decay class (Harmon & Sexton, 1996) and then grouped as either sound (classes 1 and 2) or rotten (classes 3–5) to enable comparison with data from 25 years postfire (Nelson et al., 2016).

Live aboveground lodgepole pine biomass N pools were estimated by multiplying biomass components (foliage, branches, boles) by N concentrations, either from sampled values (foliar %N) or published values (branches = 0.17% N, boles = 0.035% N; Fahey et al., 1985). Understory vegetation N pools were estimated by multiplying sampled N concentrations by the biomass of the five dominant species. Litter N pools were calculated by multiplying sampled litter mass by litter N concentrations. Nitrogen pools in downed coarse wood were estimated by multiplying biomass by published N concentrations (0.065% for decay class 1, 0.085% for decay class 2, and 0.20% for decay classes 3–5; Fahey, 1983). We conducted sensitivity analyses for all N pools estimated in this study to explore how potential measurement errors and variations in N concentrations in different biomass components may affect N pool estimates (Appendix S2).

Soil N pools in the top 15 cm of soil were calculated by multiplying fine particle (<2 mm) bulk density (hereafter: bulk density) sampled in 2023 with N concentrations sampled in 2022. Turner et al. (2019) did not measure bulk density in the field and assumed a constant bulk density of 1 g cm^−3^ for 15 and 25 years postfire. Yet, a constant bulk density does not capture declines over time or variation among stands in bulk density. Thus, we used soil bulk density values from this study, an ongoing fertilization experiment in Grand Teton National Park bordering Yellowstone to the south, and Smithwick et al. (2009) to “backcast” soil bulk density for the prior sampling times (15 and 25 years postfire, Turner et al., 2009 and 2019, respectively). We then used the backcast bulk density values (see Appendix S2 for details) to recalculate soil N pools for 15 and 25 years postfire. Therefore, estimates of total soil N at 15 and 25 years postfire used in the current analysis were 7% and 20% lower (respectively) than estimates presented in Turner et al. (2019).

### Data analysis

We analyzed data at the plot level, consistent with prior studies (Turner et al., 2009, 2019). To assess change over time (Q1) in N concentration, pools, and annual resin‐sorbed N for plots sampled at 25 and 35 years postfire, we used paired *t* tests (*n* = 14, α = 0.05) and inspected 1:1 plots. Prior to analysis, we tested for normality of differences using Shapiro–Wilk tests (α = 0.05) and transformed variables if differences were non‐normal (Tables 1 and 2). We excluded extreme outliers that remained after transformation (below first quartile +1.5 interquartile range or above third quartile +1.5 interquartile range). If pairwise differences at 25 and 35 years postfire were significant, we calculated annual rates of change for 15–25 and 25–35 years postfire for plots sampled at all three time periods (*n* = 10; as explained in Turner et al. (2019), two plots were moved at 25 years postfire due to grizzly bear closures). We then used paired *t* tests (α = 0.05) to test whether plot‐level rates of change differed between 15–25 and 25–35 years postfire. Rates of change were calculated using untransformed data but transformed prior to statistical analysis if they were non‐normal. No extreme outliers remained after transformations were applied.

**TABLE 1:** Characteristics of 14 lodgepole pine stands 15, 25, and 35 years after the 1988 Yellowstone fires.

Variable	15 years postfire^a^	25 years postfire^b^	35 years postfire^c^	*p* value	*t* value
Lodgepole pine density (stems ha^−1^)^(log10)^	84,333 (47,009)	58,605 (29,358)	39,029 (17,283)	0.072	−1.962
Aboveground live biomass (Mg ha^−1^)					
Lodgepole pine					
Foliar biomass	6.7 (4.3)	**8.4 (1.3)**	**13.0 (2.4)**	**0.003**	3.677
Bole biomass	4.6 (1.6)	**23.1 (3.8)**	**43.7 (5.2)**	**<0.001**	**5.702**
Branch biomass	2.8 (1.2)	**5.4 (0.9)**	**10.2 (1.9)**	**0.004**	**3.494**
Total lodgepole pine	14.3 (6.6)	**36.9 (5.8)**	**67.9 (9.0)**	**<0.001**	**5.184**
Aboveground net primary productivity (Mg ha^−1^ year^−1^)					
Lodgepole pine	4.5 (1.5)	**7.3 (1.2)**	**13.7 (2.6)**	**0.003**	**3.575**
Understory vegetation^(l)^	1.3 (0.21)	**1.0 (0.2)**	**0.4 (0.1)**	**<0.001**	**−5.944**
*Lupinus argenteus* ^(sqrt)^	0.01 (0.01)	**0.05 (0.02)**	**0.03 (0.01)**	**0.041**	**−2.269**
Forest floor litter mass (Mg ha^−1^)	4.4 (0.62)	**5.0 (0.9)**	**7.0 (1.0)**	**0.030**	**2.435**
Downed coarse wood biomass (Mg ha^−1^)					
Sound (decay classes 1, 2)	NA	**67.0 (9.3)**	**0 (0)**	**<0.001**	**−7.206**
Rotten (decay classes 3, 4, 5)	NA	**24.8 (4.1)**	**85.2 (11.2)**	**<0.001**	**5.207**
Total (all decay classes)	NA	91.8 (9.7)	85.2 (11.2)	0.251	−1.200

*Note*: The table shows mean and SE (in parentheses) across plots, with significant differences (paired *t* test, *p* < 0.05) between 25 and 35 years in bold. Degrees of freedom (df) for paired *t* tests are df = 13 for all variables except understory vegetation (df = 12). There were 14 plot pairs between 25 and 35 years postfire, but only 10 plot pairs between 15 and 25 years postfire due to grizzly bear closures. Superscript indicates how many extreme outliers were removed or which transformation was applied prior to paired *t* tests.

Abbreviation: NA, not applicable.

^a^ Data from Turner et al. (2009).

^b^ Data from Turner et al. (2019).

^c^ This study.

**TABLE 2:** Annual rates of change (∆) for 15–25 and 25–35 years postfire in plots sampled at all three time periods (*n* = 10; two plots were moved 25 years postfire because of grizzly bear closures).

Variable	Annual ∆, 15–25 years	Annual ∆, 25–35 years	*p* value	*t* value
Lodgepole pine				
Total aboveground biomass (Mg ha^−1^ year^−1^)	2.5 (0.4)	2.2 (0.5)	0.68	−0.42
Foliar biomass (Mg ha^−1^ year^−1^)	0.3 (0.1)	0.4 (0.2)	0.73	0.36
Foliar [N] (%N year^−1^)	**0.004 (0.002)**	**−0.01 (0.003)**	**<0.001**	**−5.99**
Foliar N pool (kg ha^−1^ year^−1^)	3.6 (1.2)	2.7 (1.5)	0.72	−0.37
Aboveground biomass N pool (kg N ha^−1^ year^−1^)	4.7 (1.4)	3.8 (1.7)	0.75	−0.33
Aboveground net primary productivity (Mg ha^−1^ year^−1^ year^−1^)	0.2 (0.1)	0.6 (0.2)	0.19	1.41
Litter and downed wood				
Litter mass (Mg ha^−1^ year^−1^)^(square root)^	0.1 (0.1)	0.1 (0.1)	0.77	0.29
Litter [N] (% N year^−1^)	0.02 (0.01)	0.01 (0.01)	0.64	−0.49
Litter N pool (kg N ha^−1^ year^−1^)	1.2 (1.0)	2.2 (0.8)	0.28	1.15
Total downed coarse wood biomass (Mg ha^−1^ year^−1^)	NA	−0.06 (0.6)	NA	NA
Total downed coarse wood N (kg ha^−1^ year^−1^)	NA	7.0 (2.3)	NA	NA
Resin‐sorbed N				
NH_4_^+^‐N (ppm bag^−1^ day^−1^ year^−1^)	**−0.20 (0.05)**	**−0.01 (0.004)**	**0.002**	**4.30**
NO_3_^−^‐N (ppm bag^−1^ day^−1^ year^−1^)^(square root)^	−0.05 (0.02)	−0.03 (0.01)	0.31	1.05
Total resin‐sorbed N (ppm bag^−1^ day^−1^ year^−1^)	**−0.25 (0.06)**	**−0.04 (0.01)**	**0.006**	**3.60**

*Note*: The sum of nitrogen (N) pool annual rates of change (lodgepole pine, litter, downed wood) presented in this table (13.0 kg N ha^−1^ year^−1^) differs from that presented in the manuscript text (15.3 kg N ha^−1^ year^−1^) because of the different sample sizes. Values represent means and SE (in parentheses) and significant differences between plots over time (paired *t* test, df = 9, *p* < 0.05) are highlighted in bold. Superscript indicates which transformation was applied prior to paired *t* tests.

To assess among‐stand variation of N pools, concentrations, and annual resin‐sorbed N (Q2), we first tested whether postfire tree regeneration continued to shape N dynamics 35 years postfire. We computed Pearson's correlations (*r*, α = 0.05) of stem density, tree biomass, and tree ANPP with N pools, concentrations, and annual resin‐sorbed N. To assess how among‐stand variation has changed over time, we compared the strength of correlations 25 and 35 years postfire. We also tested relationships between potential N fixers (silvery lupine [*Lupinus argenteus*] biomass, moss and lichen percent cover) and foliar N pools or concentrations. Finally, we tested for correlation among N pools (tree, downed wood, litter, and soil) and annual rates of change in N pools over the past decade.

Soil N pools differed widely among our 14 plots (Turner et al., 2009, 2019), and we explored this variation. We tested the relationship between soil C and N because C:N ratios are typically constrained in mineral soil (Cleveland & Liptzin, 2007; Rocci et al., 2024). We also tested the relationships of soil N with soil texture (% clay).

To assess whether measures of stand structure or stand function showed evidence of convergence (Q3), we calculated CVs at 15, 25, and 35 years postfire. All analyses were conducted in R Statistical Software (v4.4.2, R Core Team, 2024) using the segmented (Muggeo, 2025), Rstatix (Kassambara, 2025), plotrix (Lemon et al., 2025), and Tidyverse (Wickham et al., 2019) packages.

## RESULTS

### Stand development

Among the 14 plots, lodgepole pine stem density averaged 39,029 stems ha^−1^ (range 1333–207,933 stems ha^−1^) at 35 years postfire, and pairwise comparisons between 25 and 35 years postfire showed no significant differences in stem density (Table 1; Appendix S1: Figure S1). By contrast, average live lodgepole pine biomass nearly doubled from 36.9 Mg ha^−1^ at 25 years postfire to 67.9 Mg ha^−1^ at 35 years postfire (Table 1), and annual rates of change were not different between 15–25 and 25–35 years postfire (Table 2). Bole and branch biomass increased more strongly (~89% increase for both) than foliar biomass (~40% increase; Table 2). Average lodgepole pine ANPP increased from 7.3 to 13.7 Mg ha^−1^ year^−1^ in the most recent decade (Table 1), and annual rates of change in both decades were not different (Table 2). The positive relationship between ANPP and stem density among the 14 plots strengthened (*r* = 0.66, *p* = 0.01 at 25; *r* = 0.91, *p* < 0.001 at 35 years postfire; Appendix S1: Table S2).

In contrast to lodgepole pine, average understory biomass declined from 1.0 to 0.4 Mg ha^−1^ from 25 to 35 years postfire (Table 1), and rates of change were not different between 15–25 and 25–35 years postfire (Table 2). At 35 years postfire, total understory cover ranged from 0.5% to 35% (Appendix S1: Table S1). The graminoid *Carex geyeri* occurred most frequently (12/14 plots, 0.2%–7.2% cover), followed by silvery lupine (7/14 plots, 0.5%–8.2% cover) and the ericaceous shrub *Vaccinium scoparium* (7/14 plots, 0.32%–21.2% cover). The most common nonliving cover types were litter (34%–82% cover), woody material (9%–32% cover), and bare soil (1%–30% cover). Among nonvascular plants and lichens, the moss *Polytrichum juniperinum* (14/14 plots, 0.2%–14.2% cover) and the lichen *Cladonia fimbriata* (14/14 plots, 0.1%–8.4% cover) occurred most frequently, although N‐fixing lichens (*Peltigera* spp.) were also present in 10/14 plots (0.04%–1% cover).

Average forest floor litter mass, which primarily consisted of needles, increased from 5.0 to 7.0 Mg ha^−1^ since 25 years postfire. Simultaneously, downed coarse wood (primarily trees killed in the 1988 fire that have since fallen) showed evidence of advanced decomposition over the past decade (Table 1). The total mass of downed coarse wood did not decline significantly (Table 1), but no sound coarse wood (decay classes 1–2) remained. Rather, the biomass of wood at more advanced stages of decomposition (classes 3–5) more than tripled, increasing from 24.8 Mg ha^−1^ at 25 years postfire to 85.2 Mg ha^−1^ at 35 years postfire.

### Q1: Change over time

At 35 years postfire, lodgepole pine foliar N concentration averaged 0.98% N, a small but significant decline from 25 years postfire (Table 3; Appendix S1: Table S1). Average total aboveground lodgepole pine biomass N pools increased from 106.3 to 167.1 kg N ha^−1^ between 25 and 35 years postfire at a rate comparable to 15–25 years postfire (Figure 2, Table 2). The average foliar N pool increased from 89.0 to 134.4 kg N ha^−1^ and accounted for most (>80%) of the total aboveground tree biomass N pool at both 25 and 35 years postfire (Table 3). Annual rates of change in the lodgepole pine foliar N pool were not different between 15–25 and 25–35 years postfire, averaging 3.1 kg N ha^−1^ year^−1^ (Figure 2, Table 2). Bole (stem) N pools increased from 8.1 to 15.3 kg N ha^−1^ while branch N pools increased from 9.2 to 17.4 kg N ha^−1^.

**TABLE 3:** Nitrogen (N) concentrations, pools, and availability in 14 lodgepole pine stands at 25 and 35 years postfire.

Variable	25 years postfire^a^	35 years postfire	*p* value	*t* value
Aboveground live biomass				
Lodgepole pine				
Foliar [N] (%) ^(3; log10)^	**1.1 (0.03)**	**0.98 (0.02)**	**<0.001**	**−8.212**
Foliar N pool (kg N ha^−1^)	**89.0 (13.0)**	**134.4 (21.9)**	**0.009**	**3.079**
Branch N pool (kg N ha^−1^)	**9.2 (1.6)**	**17.4 (3.1)**	**0.004**	**3.493**
Bole N pool (kg N ha^−1^)	**8.1 (1.3)**	**15.3 (1.8)**	**<0.001**	**5.702**
Total biomass N pool (kg N ha^−1^)	**106.3 (15.7)**	**167.1 (26.5)**	**0.005**	**3.406**
Understory vegetation N pool (kg N ha^−1^)^b^	NA	7.4 (1.5)	NA	NA
*Lupinus argenteus* (kg N ha^−1^)	NA	0.9 (0.4)	NA	NA
Litter, downed wood, and mineral soil				
Forest floor litter				
Litter [N] (%)	**0.82 (0.03)**	**0.95 (0.03)**	**0.006**	**3.282**
N pool (kg N ha^−1^)	**39.5 (6.5)**	**68.1 (10.3)**	**0.002**	**3.959**
Downed coarse wood				
Sound biomass N pool (kg N ha^−1^)	**56.9 (7.9)**	**0 (0)**	**<0.001**	**−7.206**
Rotten biomass N pool (kg N ha^−1^)	**49.7 (8.3)**	**170.3 (22.3)**	**<0.001**	**5.207**
Total downed coarse wood (kg N ha^−1^)	**106.6 (10.5)**	**170.3 (22.3)**	**0.002**	**3.833**
Total aboveground N pool (kg N ha^−1^)^c^	**252.4 (16.5)**	**405.4 (37.5)**	**<0.001**	**5.464**
Mineral soil (0–15 cm)				
Mineral soil [N] (%)	0.08 (0.01)	0.09 (0.01)	0.206	1.332
Total soil N (kg ha^−1^) ^(log10)^	948.1 (87.9)	985.9 (78.3)	0.47	0.739
Annual resin‐sorbed N				
NH_4_^+^‐N (ppm bag^−1^ day^−1^)	**0.22 (0.05)**	**0.14 (0.04)**	**0.012**	**−2.938**
NO_3_^−^‐N (ppm bag^−1^ day^−1^)	**0.47 (0.12)**	**0.13 (0.04)**	**0.002**	**−3.821**
Total N (ppm bag^−1^ day^−1^)	**0.69 (0.17)**	**0.27 (0.08)**	**0.002**	**−3.836**

*Note*: Values presented are mean and SE (in parentheses), and significant differences between plots over time (paired *t* test, *p* < 0.05) are bold. Paired *t* test degrees of freedom are df = 13 for all variables except foliar [N] (df = 10). Superscript indicates how many extreme outliers were removed or which transformation was applied prior to paired *t* tests.

Abbreviation: NA, not applicable.

^a^ Data from Turner et al. (2019).

^b^ N pool was computed for the five most dominant (by % cover) vascular understory species per plot (Appendix S1: Table S1). These species account for 55%–100% (mean = 88%) of total understory biomass per plot.

^c^ Sum of aboveground N pools measured at 25 and 35 years postfire (lodgepole pine, downed coarse wood, forest floor litter).

**FIGURE 2:**
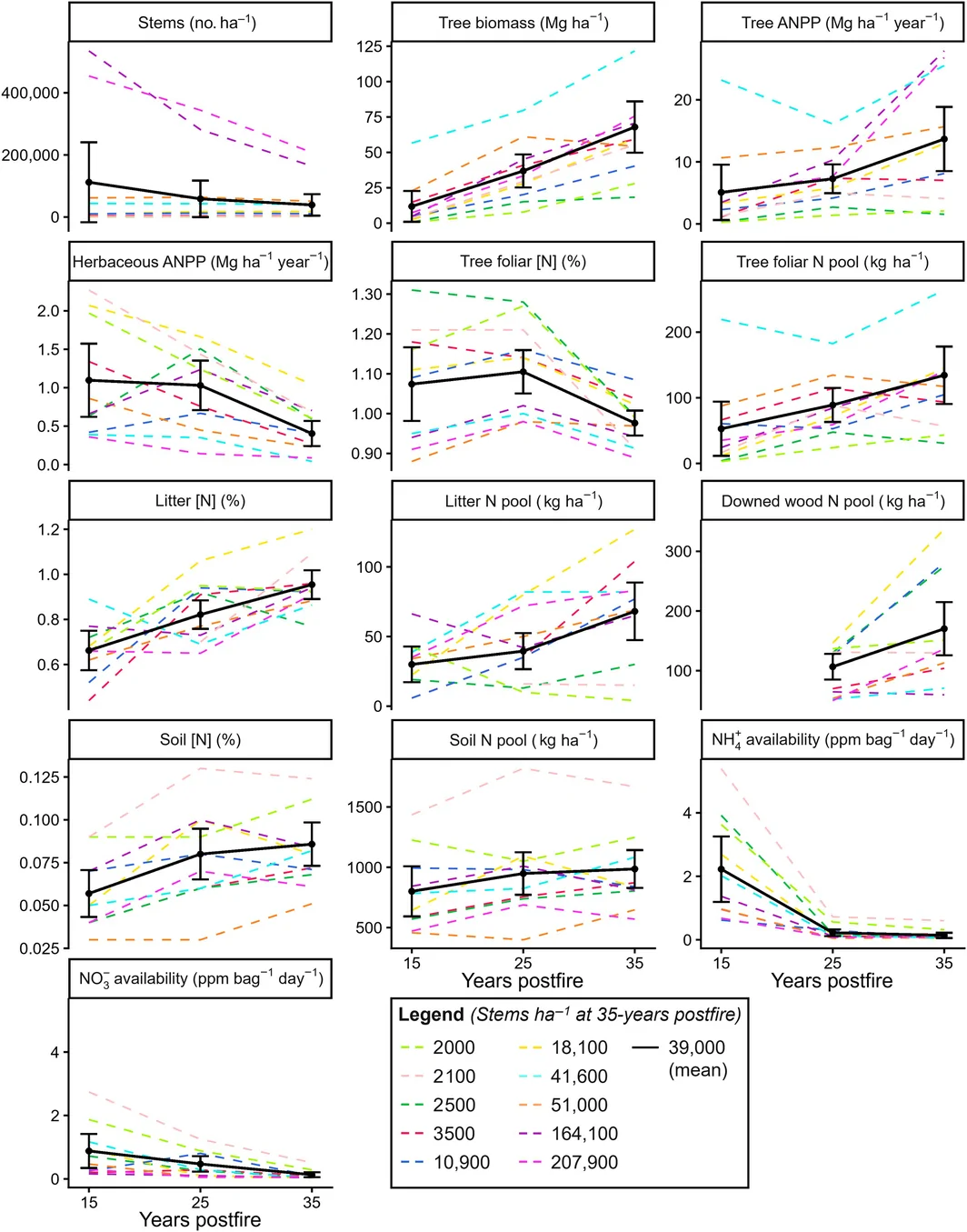
Plot‐level (dashed lines) and mean (solid lines, ±2 SE) trajectories of lodgepole pine stem density, aboveground net primary productivity (ANPP), and biomass as well as nitrogen (N) pools, concentrations, and availability in plots sampled at 15, 25, and 35 years postfire (*n* = 10; two plots had to be moved 25 years postfire due to grizzly bear closures). Trends over time display persistent variation among stands regenerating from the 1988 Yellowstone fires. Note that the *y*‐axis differs among panels and units are included in panel headings.

Forest floor litter N concentrations averaged 0.95% N at 35 years postfire compared to 0.82% N at 25 years postfire (Table 3). The forest floor litter N pool increased from 39.5 to 68.1 kg N ha^−1^ from 25 to 35 years postfire (Table 3). Annual rates of change of the litter N pool were not different between the decades, increasing by an average of 1.7 kg N ha^−1^ year^−1^ (Figure 2, Table 2). Total downed wood N pools increased from 106.6 to 170.3 kg N ha^−1^.

On average, total aboveground N pools measured at 25 and 35 years postfire (lodgepole pine, litter, and downed wood) grew at a rate of 15.3 kg N ha^−1^ year^−1^ from 252.4 to 405.4 kg N ha^−1^ (Figure 3). Among stands, the annual rate of change in aboveground N pools varied from −2.5 to 32.9 kg N ha^−1^ year^−1^ between 25 and 35 years postfire (Appendix S1: Figure S2f).

**FIGURE 3:**
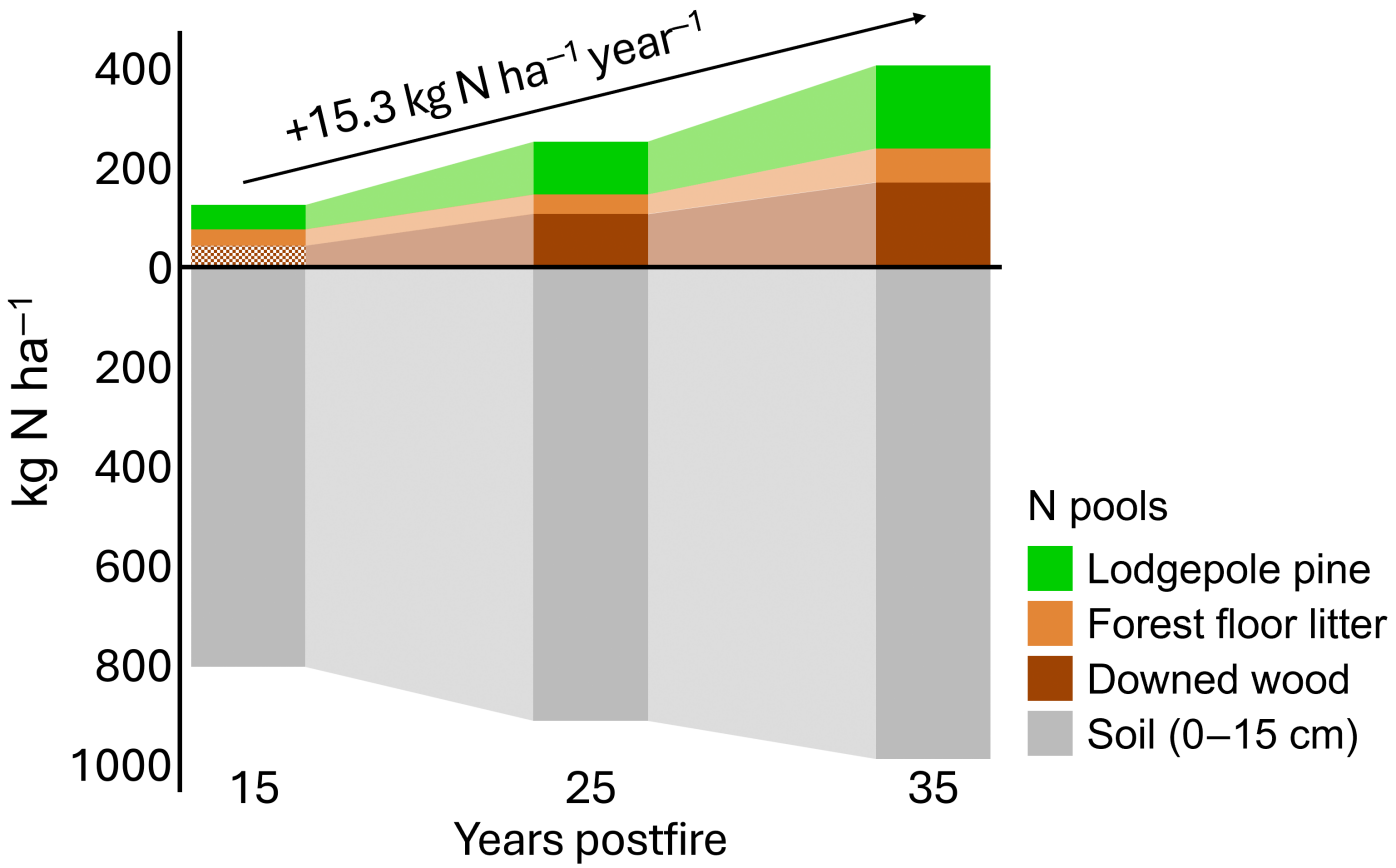
Trajectories of ecosystem nitrogen (N) pools from 15 to 35 years postfire. Average aboveground N pools increased by 15.3 kg N ha^−1^ year^−1^ from 15 to 35 years postfire while soil N pools did not change. Note that the *y*‐axis runs in two directions from the 0‐intercept, and that downed wood at 15 years postfire is shaded differently because it was not measured.

Mineral soil (0–15 cm) N concentrations averaged 0.09% at 35 years postfire and were not different at 25 years postfire (Table 3). The total soil N pool averaged 985.9 kg N ha^−1^ at 35 years postfire and remained unchanged from 25 years postfire (Table 3, Figures 2 and 3; Appendix S1: Figure S1). Average annual resin‐sorbed N declined from 0.69 to 0.27 ppm bag^−1^ day^−1^ between 25 and 35 years postfire (Table 3). Nitrate‐N accounted for most of the decline, dropping from 0.47 to 0.13 ppm bag^−1^ day^−1^ between 25 and 35 years postfire while ammonium‐N declined from 0.22 to 0.14 ppm bag^−1^ day^−1^ over the same period. Annual rates of change for ammonium‐N and total resin‐sorbed N were greater from 15 to 25 than from 25 to 35 years postfire (Table 2).

### Q2: Spatial variation

Among the 14 stands at 35 years postfire, live aboveground lodgepole pine biomass N pools ranged from 39.2 to 377.2 kg N ha^−1^ and were positively correlated with lodgepole pine ANPP (*r* = 0.77, *p* = 0.001) and biomass (*r* = 0.96, *p* < 0.001), similar to relationships at 25 years postfire (Figure 4; Appendix S1: Table S2, Figures S3 and S4). There was no relationship between live biomass N and stem density (Figure 4; Appendix S1: Table S2, Figure S5). Litter N pools ranged from 4.0 to 127.0 kg N ha^−1^ at 35 years postfire and were positively correlated with lodgepole pine aboveground biomass (*r* = 0.58, *p* = 0.03; Figure 4; Appendix S1: Figure S4). The downed wood N pool varied from 59.6 to 337.0 kg N ha^−1^ among stands, but prior relationships at 25 years postfire with ANPP, stem density, and aboveground biomass were no longer significant (Figure 4; Appendix S1: Table S2). When live and detrital aboveground N pools were summed, total aboveground N ranged from 211.7 to 638.5 kg N ha^−1^ at 35 years postfire and was related to lodgepole pine aboveground biomass (*r* = 0.62, *p* = 0.02, *n* = 14) but not stem density or ANPP. At 25 years postfire, total aboveground N pools were positively correlated with lodgepole pine ANPP (*r* = 0.70, *p* = 0.01, *n* = 14) and aboveground biomass (*r* = 0.76, *p* = 0.002, *n* = 14; Appendix S1: Figures S6–S8).

**FIGURE 4:**
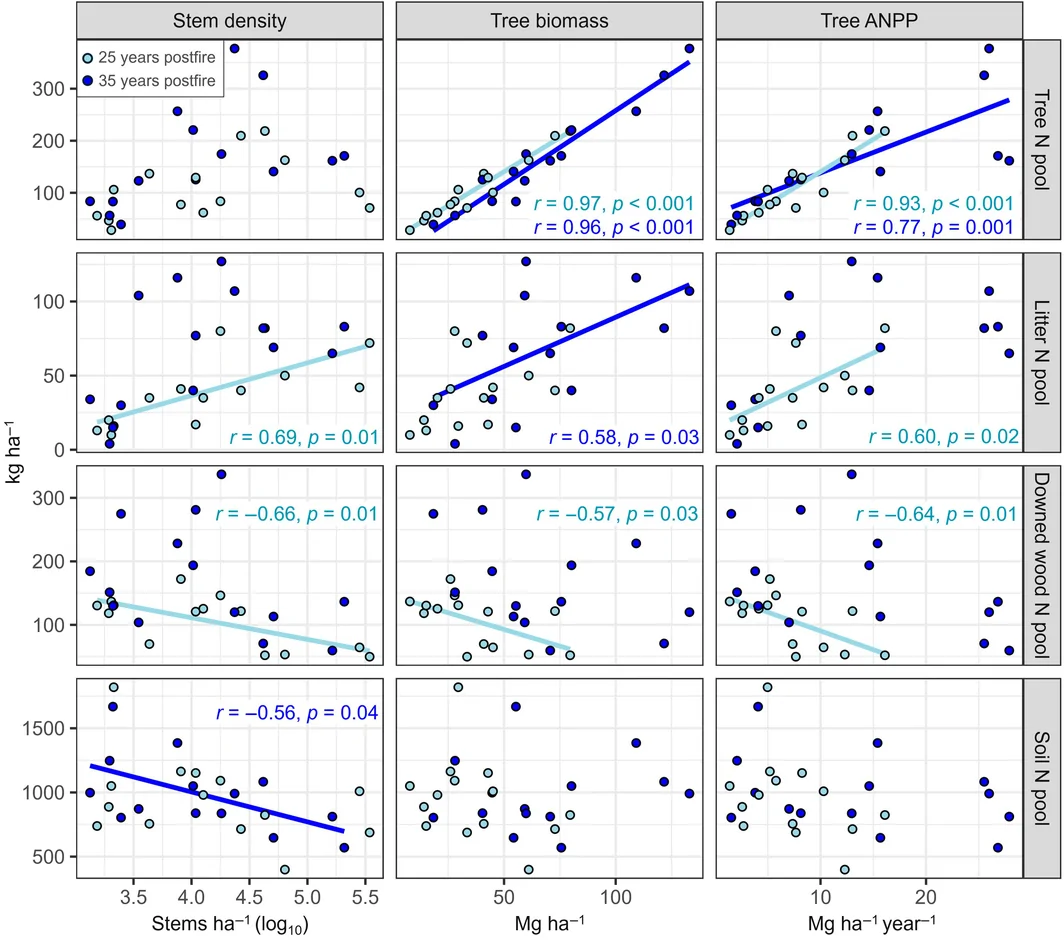
Pearson correlations (*r*) of lodgepole pine stem density, aboveground biomass, and aboveground net primary productivity (ANPP) with ecosystem nitrogen (N) pools at 25 (light blue) and 35 (dark blue) years postfire. Trendlines are included for significant correlations (*p* < 0.05, *n =* 14), and the color of the trendline corresponds to the time postfire at which the significant correlation was observed.

Soil N pools (0–15 cm) ranged from 570 to 1668 kg N ha^−1^ at 35 years postfire and were negatively correlated with lodgepole pine stem density (*r* = −0.56, *p* = 0.04, *n* = 14) but unrelated to ANPP or aboveground biomass (Figure 4; Appendix S1: Table S2, Figures S3–S5). Ten years prior, soil N pools were unrelated to lodgepole pine stem density, ANPP, and aboveground biomass (Figure 4). Soil N pools were positively correlated with percent clay (*r* = 0.60, *p* = 0.01, *n* = 14). Soil [C] and [N] were strongly correlated (*r* = 0.98, *p* < 0.001, *n* = 14; Appendix S1: Table S3) with soil C:N ratios ranging from 22.6 to 31.4 (mean = 26.2).

Total resin‐sorbed N ranged from 0.06 to 1.12 ppm bag^−1^ day^−1^ and was unrelated to lodgepole pine stem density, ANPP, and aboveground biomass at 35 years postfire (Appendix S1: Table S2, Figures S3–S5). Ten years prior, total resin‐sorbed N was negatively related to lodgepole pine stem density (*r* = −0.68, *p* = 0.01, *n* = 14). Resin‐sorbed ammonium varied from 0.03 to 0.60 ppm bag^−1^ day^−1^ among stands and was unrelated to lodgepole pine stem density, ANPP, and aboveground biomass at 35 years postfire but negatively related to all at 25 years postfire. Resin‐sorbed NO_3_^−^ varied from 0.03 to 0.52 ppm bag^−1^ day^−1^ and was negatively correlated with lodgepole pine ANPP (*r* = −0.55, *p* = 0.04, *n* = 14). Ten years earlier, resin‐sorbed NO_3_^−^ was negatively correlated with lodgepole pine stem density (*r* = −0.69, *p* = 0.01, *n* = 14).

Understory vegetation aboveground live N pools were two orders of magnitude lower than aboveground live tree N pools and one order of magnitude lower than litter N pools (Table 3). Although understory N concentrations were higher compared to lodgepole pine foliage, understory biomass was very low (Table 1). The understory N pool was not correlated with either resin‐sorbed N or total soil N.

There were some relationships between putative N fixers (e.g., silvery lupine, moss, lichen) and N concentrations (Appendix S1: Figure S9). Silvery lupine biomass ranged from 0 to 152 kg ha^−1^ and was positively correlated with lodgepole pine foliar N concentrations (*r* = 0.55, *p* = 0.04, *n* = 14). Moss cover, which ranged from 0.2% to 14.2%, was also positively correlated with lodgepole pine foliar N concentrations (*r* = 0.66, *p* = 0.01, *n* = 14). Lichen cover ranged from 0.1% to 15.0% and was negatively correlated with soil N pools (*r* = −0.58, *p* = 0.03, *n* = 14) but not correlated with lodgepole pine foliar N concentrations. However, there were few relationships among the N pools we quantified (Appendix S1: Table S4, Figures S10 and S11), except for a positive correlation of aboveground lodgepole pine biomass N pools and litter N pools (*r* = 0.63, *p* = 0.002, *n* = 14). There were also no significant relationships among annual rates of change in N pools from 25 to 35 years postfire (e.g., soil N pools did not decline as aboveground N pools increased; Appendix S2: Table S4, Figure S2).

### Q3: Time × space

Measures of structure had higher among‐stand variation than measures of function at 35 years postfire but evidence of convergence over time was more consistent in measures of structure (Figure 5, Table 4). Among‐stand variation (CV) in lodgepole pine stem density declined from 182% to 164% between 15 and 25 years postfire to 147% by 35 years postfire (Table 4). Variation in aboveground tree biomass declined sharply from 15 to 25 years postfire (145%–60%) and further declined to 49% by 35 years postfire. Simultaneously, variation in ANPP declined from 139% to 62% from 15 to 25 years postfire but increased again to 79% by 35 years postfire (Table 4). Foliar, litter, and soil N concentrations, soil N pools, and herbaceous ANPP had relatively low CVs that changed little from 15 to 35 years postfire (all <42%; Table 4). Litter N pools had higher variability (~60%) that remained relatively constant over time (Figure 5, Table 4). Variability in the lodgepole pine foliar N pool declined from 130% to 55% between 15 and 25 years postfire and remained relatively constant (59%) by 35 years postfire (Table 4). Conversely, variability in resin‐sorbed N increased between 15 and 35 years postfire, from 73% to 108% for ammonium and from 96% to 120% for nitrate (Table 4), while mean values declined steeply during that time (Figure 2).

**FIGURE 5:**
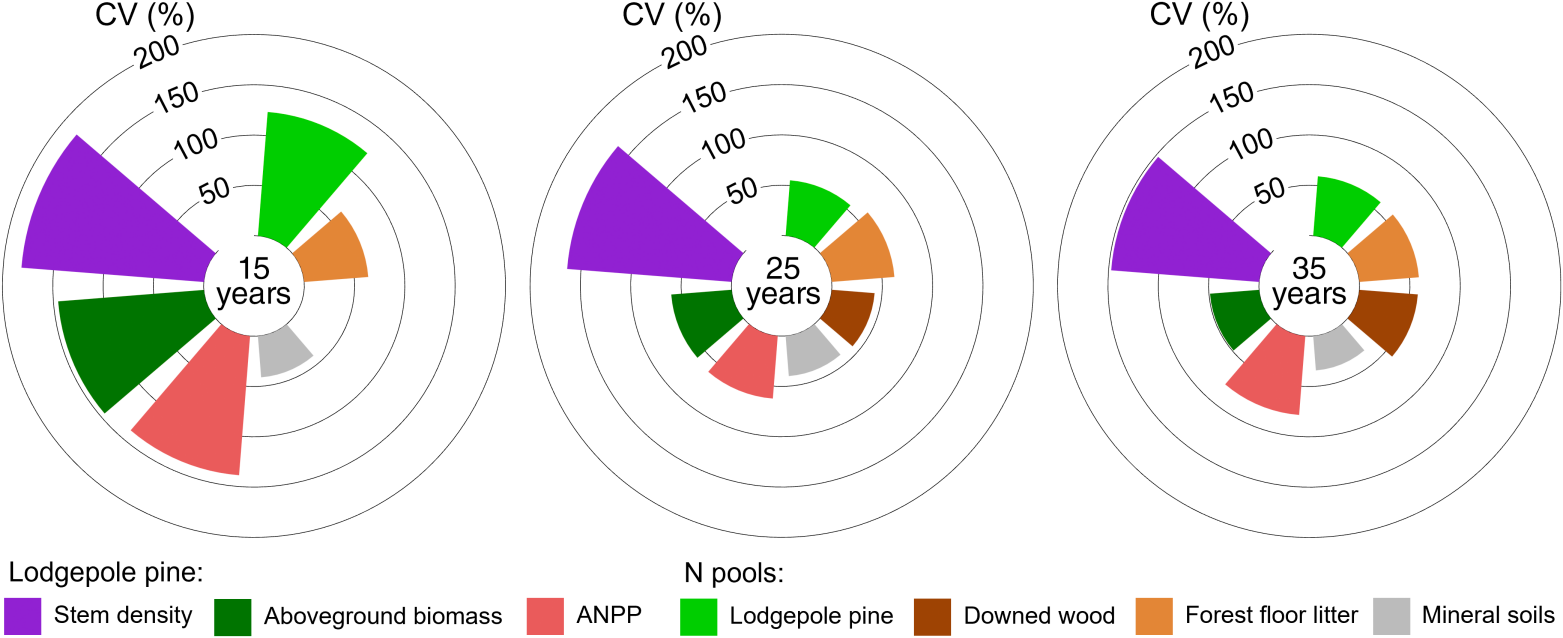
CVs (in percentage) for stand characteristics (lodgepole pine stem density, aboveground biomass, and aboveground net primary productivity [ANPP]) and nitrogen (N) pools at 15, 25, and 35 years postfire. Declining CVs (from left to right) suggest that variables are converging over time and vice versa.

**TABLE 4:** CVs (in percentage) for structural (stem density, aboveground tree biomass) and functional response variables (aboveground net primary productivity [ANPP], nitrogen [N] concentrations, N pools, and annual resin‐sorbed N) at 15, 25, and 35 years postfire.

Variable	CV (%), 15 years postfire	CV (%), 25 years postfire	CV (%), 35 years Postfire
Stand structure and productivity			
Lodgepole pine stem density	182	164	147
Lodgepole pine biomass	145	60	49
Lodgepole pine ANPP	139	62	79
Herbaceous ANPP	69	57	68
Litter, downed wood, and mineral soil			
Foliar [N]	14	10	6
Foliar N pool	124	55	59
Lodgepole pine aboveground N pool	124	55	59
Litter [N]	20	17	13
Litter N pool	64	62	59
Downed wood N pool	NA	43	58
Soil [N]	38	36	28
Soil N pool	41	40	34
Resin‐sorbed N			
NH_4_^+^‐N	73	99	108
NO_3_^−^‐N	96	103	120
Total N	77	100	113

## DISCUSSION

Our study highlights that N pools continue to recover and young lodgepole pine forests remain highly productive 35 years postfire while considerable variation among stands persists. Aboveground N pools in lodgepole pine biomass, forest floor litter, and downed coarse wood increased steadily from 15 to 35 years after high‐severity fire, but rates of increase in N pools had not yet slowed down and were not different in both decades postfire. Within just 35 years, young lodgepole pines had almost fully recovered their pre‐fire N pools, as the mean aboveground lodgepole pine biomass N pool was 95% of the average across a 300‐year lodgepole pine chronosequence (Smithwick et al., 2009). Yet, the rapid recovery of aboveground N occurred without commensurate declines in surface (0–15 cm) soil N pools and despite extremely low annual resin‐sorbed NH_4_^+^ and NO_3_^−^. Among the 35‐year‐old stands, lodgepole pine biomass N pools were positively correlated with aboveground tree biomass and productivity but not stem density. Thus, the enormous initial variation in postfire tree establishment driven by prefire serotiny and burn severity (Turner et al., 1994, 1997) continued to affect N dynamics. This influence on N is now manifest via variation in tree biomass and productivity since stem density was no longer directly related to N dynamics. Our data also suggest that some functional response variables (e.g., lodgepole pine ANPP) are not yet converging, as CVs largely remained similar over the past decade. Our data highlight changing but persistent effects of disturbance legacies on N pools as postfire stand development proceeds, but do not resolve the sources of recovered N.

### Change over time

Aboveground lodgepole pine biomass and productivity increased at similar rates from 15 to 35 years postfire, suggesting that stands have not yet reached peak productivity. This is consistent with studies that estimate peak ANPP in lodgepole pine forests between 40 and 70 years postfire (Kashian et al., 2013; Olsson et al., 1998; Pearson et al., 1987). The aboveground tree biomass N pool increased in tandem with increases in aboveground foliar biomass that offset the small, but significant declines in foliar N concentrations from 25 to 35 years postfire (1.10%–0.98% N). Declining foliar N concentrations likely reflect a dilution effect, and we recognize that foliar nutrient concentrations may not be reliable indicators of plant nutrient supply, demand, or limitation (Binkley et al., 2025; Jarrell & Beverly, 1981).

Litter and downed coarse wood N pools increased alongside live aboveground N pools. The increase in litter mass mirrored the increase in lodgepole pine foliar biomass over the past decade, while the increase in litter N concentrations is likely due to saprotrophic fungi acquiring N from soils (Fahey et al., 1985; Remsburg & Turner, 2006; Yavitt & Fahey, 1986). Nutrient resorption efficiency of lodgepole pines is generally high but declines over time, which could also increase litter N concentrations (Bothwell et al., 2001; Prescott et al., 1989). Increases in the downed coarse wood N pool likely reflect changes in N concentrations and mass associated with decay stage. Nearly all fire‐killed snags had fallen by 15 years postfire (Kiel, Romme, & Turner, 2023), but many remain elevated off the soil for years (Remsburg & Turner, 2006). Large, fallen fire‐killed trees begin to decompose slowly only after they come in contact with soil. During decomposition, N concentrations increase in part due to microbial respiration and loss of C (Boddy & Watkinson, 1995), but also due to microbial N fixation and N translocation from soils (Laiho & Prescott, 1999; Rinne et al., 2017). Our results show that N continues to accumulate in litter and downed wood pools 35 years postfire, consistent with postfire N dynamics in lodgepole pine forests inferred from lake sediment cores that suggest detrital N pools recover between 50 and 75 years postfire (Dunnette et al., 2014).

Counter to our expectations, increasing aboveground N pools were not paralleled by declines in surface soil N pools. Theory suggests that mineral soil N pools initially decline as aggrading trees accumulate biomass and increase after tree productivity (and thus tree N demand) declines (i.e., Lovett et al., 2018; Rastetter et al., 2021). Yet, surface mineral soil N pools in Yellowstone did not change during a period of high tree productivity from 25 to 35 years postfire. This is consistent with other studies that report no change (Giesen et al., 2008) or slight increases (Hupperts et al., 2025; Shen et al., 2025) in soil N pools after stand‐replacing fire. Some methodological considerations may be important when interpreting these results. First, we only sampled soils to a depth of 15 cm despite the potential importance of deep soil N dynamics (Almaraz et al., 2025; Heumann & Cleveland, 2026). Second, soil N pools are spatially heterogeneous and prone to measurement error (Huntington et al., 1988), making it difficult to detect small changes over time. Yet, over the past decade, mean aboveground N pools increased by 15.5% (~153 kg N ha^−1^) of the mean soil N pool at 35 years postfire, but a corresponding decline in soil N pools would be near the minimum detectable difference of ~153 kg N ha^−1^ (following Huntington et al. (1988) who suggested that the minimum detectable difference of soil N pools would be estimated by multiplying the SE of soil N pools by 1.96). Although our findings do not suggest that mineral soil N subsidized replenishment of aboveground N pools, methodological limitations should be considered when interpreting our results.

Resin‐sorbed NH_4_^+^ and NO_3_^−^ declined over the past decade, but the rate of decline of NH_4_^+^ availability was lower in the recent decade relative to 15–25 years postfire, when available N declined rapidly. Brief pulses of increased N availability after stand‐replacing fire are commonly followed by rapid immobilization as biological demand increases (Gustine et al., 2022). In Yellowstone, N consumption exceeded gross N production as soon as 1–4 years postfire (Turner et al., 2007). The lack of association between resin‐sorbed (plant‐available) N and lodgepole pine productivity or biomass N pools is also consistent with the pines relying on fungal symbionts that can access soil organic N (Bothwell et al., 2001; Nicolás et al., 2019). Where the N acquired by fungal symbionts originates from remains unclear, given that we found no relationship between soil and aboveground N pools as well as no evidence of the N‐fixing tuberculate ectomycorrhizal fungi reported for young lodgepole pines in British Columbia (Chapman & Paul, 2012).

### Spatial variation

Among stands, we found that effects of postfire tree regeneration density on N dynamics are now manifest indirectly through effects on tree biomass and productivity. Lodgepole pine ANPP and biomass remained strongly correlated with live tree N pools, but stem density was no longer related to any aboveground N pools or concentrations. Stem density was negatively related to soil N pools at 35 but not 25 years postfire, and this is likely attributable to variation in soil mineralogy, texture, and bulk density. Finer textured soils hold more N (Rocci et al., 2024), but lodgepole pine somewhat prefers coarse textured soils (Koch, 1996; Stephens, 1966). Further, bulk density declines with increasing stem density due to the greater abundance of fine roots, thus lowering estimates of soil N pools.

The origins of N accumulating in aboveground pools remain uncertain. All the aboveground N pools we measured acquire at least some N from soil pools, but we detected no decline in surface soil N pools that could explain increasing aboveground pools. Therefore, N moving from surface soils to aboveground pools must be resupplied at similar rates from a combination of unknown sources. Biological N fixation and/or movement of N from deep soils likely play important roles. Positive relationships between foliar N concentration and silvery lupine biomass and moss cover suggest that N inputs via biological fixation could provide a new source of N to the ecosystem. Yet, measured inputs from symbiotic fixation, assuming all N in silvery lupine in our plots comes from fixation (mean 0.9, range 0.0–6.3 kg N ha year^−1^), and cryptic fixation (mean 0.26, range 0.07–2.9 kg N ha^−1^ year^−1^; Heumann et al., 2025) are insufficient to explain the increases in aboveground N pools over time (+15.3 kg N ha^−1^ year^−1^). Endophytic N fixation may play a role, but estimated N inputs from conifer endophytes are also very low (0.01–0.02 kg N ha^−1^ year^−1^, Moyes et al., 2016). Our team previously measured endophytic N fixation following the same methods as Moyes et al. (2016) but nitrogenase activity was below detection limits (Heumann et al., 2025). Deposition is another potential N source, but deposition in Yellowstone is also very low (2–3 kg ha^−1^ year^−1^, Hoffman et al., 2019; National Atmospheric Deposition Program, 2025). The role of soil N dynamics deeper than 30 cm (as measured by Smithwick et al., 2009) could be key to balancing the N budget in young lodgepole pine forests (Heumann & Cleveland, 2026).

### Convergence among stands

Our finding that among‐stand variation in measures of ecosystem function declined more rapidly than measures of stand structure aligns with prior studies documenting recovery of C and N pools (function) within a century and convergence in stand density (structure) within ~200 years (Kashian et al., 2013; Kashian, Turner, & Romme, 2005; Kashian, Turner, Romme, & Lorimer, 2005; Smithwick et al., 2009). Counter to our expectations, variation in ANPP increased slightly over the last 10 years, which is largely attributable to strong increases in productivity in the two densest plots (in which stem density declined) but little change in productivity in the other plots (Figure 2). Peak rates of ANPP should be greater and occur sooner in denser relative to sparser stands (Kashian et al., 2013; Turner, 2010). Stands eventually converge in productivity within ~130 years postfire as dense stands self‐thin and sparser stands infill (Kashian, Turner, Romme, & Lorimer, 2005). Overall, much among‐stand variation remained after 35 years, suggesting a persistent legacy of initial patterns of postfire tree regeneration even as ecosystem function is recovered. As stands continue to converge in structure, N dynamics are increasingly shaped by patterns of tree biomass rather than by the variation in stem density, which still ranged from 1333 to 207,933 stems ha^−1^ at 35 years postfire relative to 533 to 535,000 stems ha^−1^ at 15 years postfire (Turner et al., 2009).

## CONCLUSIONS

Changes in N pools over 35 years of postfire stand development suggest that Yellowstone's young lodgepole pine forests remain strong N sinks. High‐severity, stand‐replacing fires drive substantial N losses and disrupt fluxes (e.g., mineralization, nitrification, and assimilation, among others) associated with internal N cycling (Gustine et al., 2022). In Yellowstone, forests retained much of the remaining postfire N with minimal postfire losses to surface waters and rapid immobilization by microbes and N assimilation by resprouting perennial vegetation (Metzger et al., 2006; Romme et al., 2009; Turner et al., 2007). Consistent with our expectations, aboveground N pools continued to recover rapidly 35 years after the fires, and this recovery is strongly shaped by among‐stand variation in tree biomass and productivity. However, counter to our expectations, recovery of aboveground N pools does not appear constrained by surface soil N dynamics or patterns of symbiotic N fixation over time or among stands. Future work exploring the role of cryptic N fixation as succession continues, and expanding the scope of N pools measured (e.g., to deeper soil) could elucidate the sources of accumulating N in young lodgepole pine forests. Overall, ecosystem function is recovering quickly relative to Yellowstone's historical fire‐return intervals, highlighting that these forests are well adapted to their historical fire regimes. Yet, variability induced by the 1988 wildfires continues to shape ecosystem function across the landscape, highlighting the importance of accounting for disturbance legacies in studies of ecosystem function.

## CONFLICT OF INTEREST STATEMENT

The authors declare no conflicts of interest.

## Supporting information


Appendix S1.

Appendix S2.

## Data Availability

Data (Keller et al., 2026) are available in the Environmental Data Initiative at https://doi.org/10.6073/pasta/5728a1743dcb34e3c9d2e2c053eda63c.
