# Supplementary material for: Nitrogen dynamics 35 years after stand‐replacing fire: Aboveground pools grow as surface soil pools remain unchanged
Source: Ecology. 2026 Oct 6;107(10):e70546. doi: 10.1002/ecy.70546 (PMC13640499; doi:10.1002/ecy.70546)
Supplement: Supplementary file 1 [file ECY-107-e70546-s001.pdf]

Appendix S1: Supplemental tables and figures

Nitrogen dynamics 35 years after stand-replacing fire: Aboveground pools grow as surface soil pools remain unchanged

Timon T. Keller, Cory C. Cleveland, Robert E. Heumann, Arielle C. Link, Lucille T. McGuire, and Monica G. Turner

Ecology

Table S1. Percent cover and aboveground net primarily productivity (ANPP) of the five most abundant vascular understory species in each plot. Some plots had < 5 understory species.

Plot name	Species	Cover (%)	ANPP (kg ha^{-1})	Percent of vascular understory cover	Percent of vascular understory ANPP
**84 Blowdown**	*Lupinus argenteus*	8.2	152.1	83.7	56.2
	Carex geyeri	1.2	96.7	12.2	35.7
	Carex rossi	0.2	19.4	2.4	7.1
	Fragaria virginiana	0.1	1.7	1.2	0.6
	Antennaria microphylla	0.0	1.0	0.4	0.4
	**Plot total**	9.8	270.8	100.0	100.0
**Biscuit Basin**	*Carex geyeri*	0.5	38.7	100.0	100.0
	**Plot total**	0.5	38.7	100.0	100.0
**Cascade Creek**	*Carex geyeri*	4.2	83.9	28.5	31.1
	Carex rossi	3.3	5.2	22.5	1.9
	Eurybia merita	1.8	27.3	12.3	10.1
	Arnica cordifolia	1.4	0.1	9.9	0.0
	Lupinus argenteus	1.4	33.3	9.9	12.3
	**Plot total**	12.1	149.8	83.0	55.5
**Cascade Meadows North**	*Vaccinium scoparium*	15.2	448.6	47.4	44.8
	Antennaria rosea	3.0	43.2	9.4	4.3
	Hieracium cynoglossoides	1.8	20.3	5.6	2.0
	Luzula sp.	1.6	35.4	5.0	3.5
	Chamerion angustifolium	1.5	45.2	4.7	4.5
	**Plot total**	23.1	592.8	72.1	59.2
**Cascade Meadows South**	*Vaccinium scoparium*	21.2	619.8	60.4	56.3
	Carex geyeri	6.1	259.1	17.5	23.5
	Chamerion angustifolium	3.1	79.9	8.9	7.3
	Arnica cordifolia	1.8	6.3	5.1	0.6
	Calamagrostis canadensis	1.6	77.5	4.7	7.0
	**Plot total**	33.9	1042.6	96.6	94.8

Plot name	Species	Cover (%)	ANPP (kg ha^{-1})	Percent of vascular understory cover	Percent of vascular understory ANPP
**Cygnet Lake North**	*Vaccinium scoparium*	8.3	252.3	83.2	53.7
	Lupinus argenteus	0.5	12.0	5.2	2.6
	Collinsia parviflora	0.3	3.1	2.8	0.7
	Carex geyeri	0.2	19.4	2.4	4.1
	Campanula rotundifolia	0.2	122.1	2.0	26.0
	**Plot total**	9.6	408.8	95.6	87.0
**Firehole Loop South**	*Lupinus argenteus*	1.3	29.6	38.1	10.1
	Carex geyeri	0.7	58.0	21.4	19.7
	Antennaria microphylla	0.4	8.6	10.7	2.9
	Eurybia merita	0.3	11.2	8.3	3.8
	Campanula rotundifolia	0.2	122.1	6.0	41.5
	**Plot total**	2.8	229.5	84.5	78.0
**Fountain East**	*Calamagrostis rubescens*	23.8	555.7	76.4	71.5
	Solidago sp.	1.3	75.3	4.1	9.7
	Arnica cordifolia	1.2	17.8	3.7	2.3
	Lupinus argenteus	1.2	26.8	3.7	3.5
	Campanula rotundifolia	1.0	27.4	3.1	3.5
	**Plot total**	28.4	703.0	91.0	90.5
**Gibbon Falls**	*Carex geyeri*	0.3	22.6	53.9	55.5
	Arctostaphylos uva-ursi	0.1	13.0	23.1	31.9
	Antennaria microphylla	0.1	1.9	15.4	4.7
	Carex rossi	0.0	3.2	7.7	7.9
	**Plot total**	0.5	40.7	100.0	100.0
**Gravel Pit**	*Calamagrostis rubescens*	1.2	67.1	74.4	76.8
	Arctostaphylos uva-ursi	0.4	20.3	25.6	23.2
	**Plot total**	1.6	87.4	100.0	100.0

Plot name	Species	Cover (%)	ANPP (kg ha^{-1})	Percent of vascular understory cover	Percent of vascular understory ANPP
**Lewis Canyon**	*Vaccinium scoparium*	6.7	206.6	41.8	29.0
	Carex geyeri	6.5	291.3	40.3	40.9
	Anaphalis margaritacea	1.3	73.9	8.0	10.4
	Hieracium albiflorum	0.7	12.3	4.2	1.7
	Chamerion angustifolium	0.3	19.3	2.0	2.7
	**Plot total**	15.5	603.4	96.3	84.7
**Pitchstone HOBO**	*Carex geyeri*	7.2	355.6	35.0	45.4
	Carex rossi	5.0	159.0	24.3	20.3
	Vaccinium scoparium	3.8	123.3	18.5	15.7
	Lupinus argenteus	2.7	15.5	13.0	2.0
	Achnatherum sp.	1.1	36.2	5.3	4.6
	**Plot total**	19.8	689.6	96.1	87.9
**Riddle Lake HOBO**	*Carex geyeri*	4.1	80.3	58.5	40.5
	Lupinus argenteus	1.4	31.5	19.3	15.9
	Vaccinium scoparium	0.6	32.0	8.5	16.1
	Carex rossi	0.4	35.5	6.3	17.9
	Chamerion angustifolium	0.3	19.3	4.6	9.7
	**Plot total**	6.8	198.5	97.2	100.0
**South Lewis Canyon**	*Vaccinium scoparium*	11.6	347.0	66.3	58.3
	Carex geyeri	5.8	234.1	33.3	39.3
	Chamerion angustifolium	0.1	14.1	0.5	2.4
	**Plot total**	17.6	595.2	100.0	100.0

Figure S1 (caption on following page)

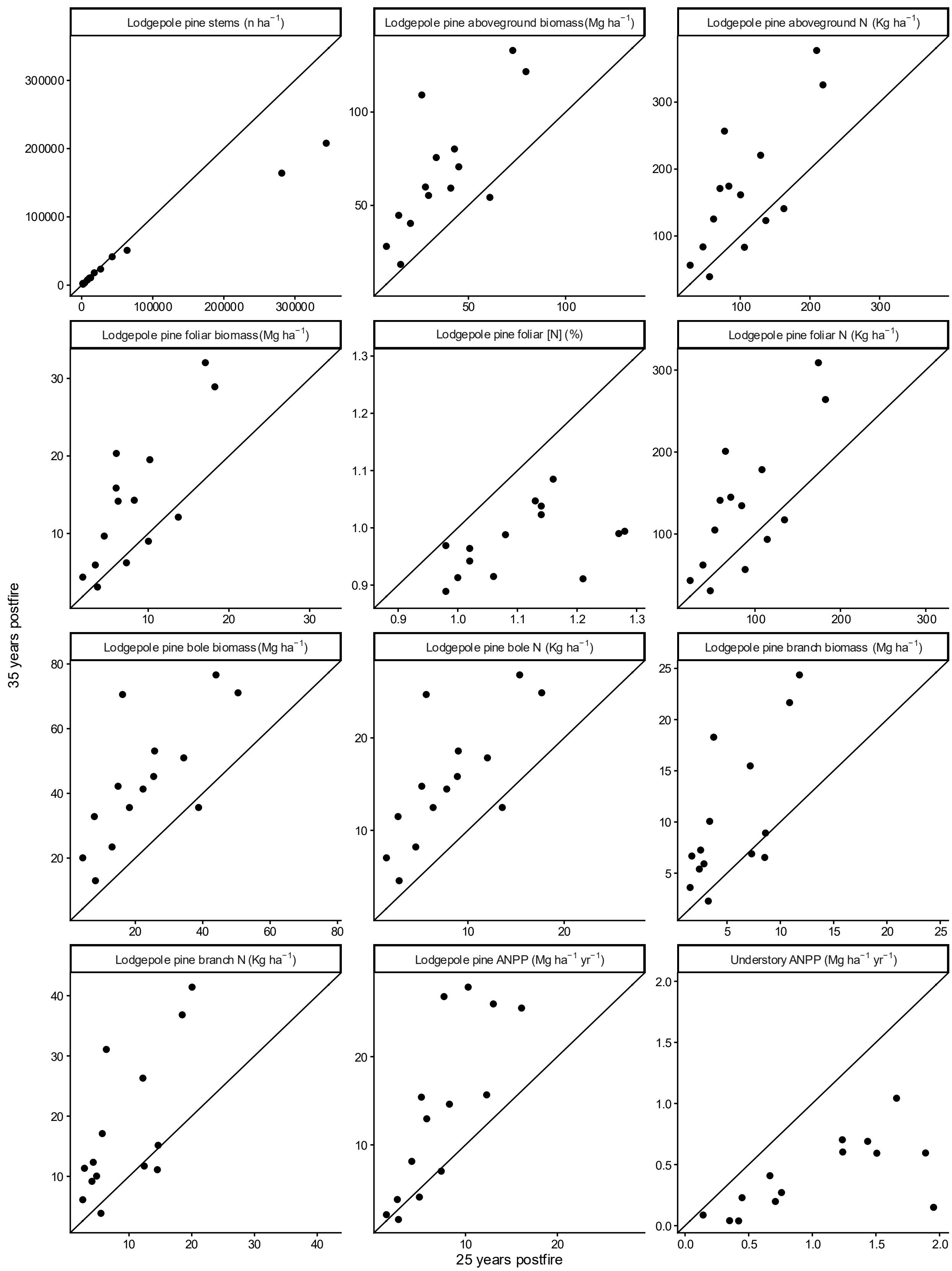

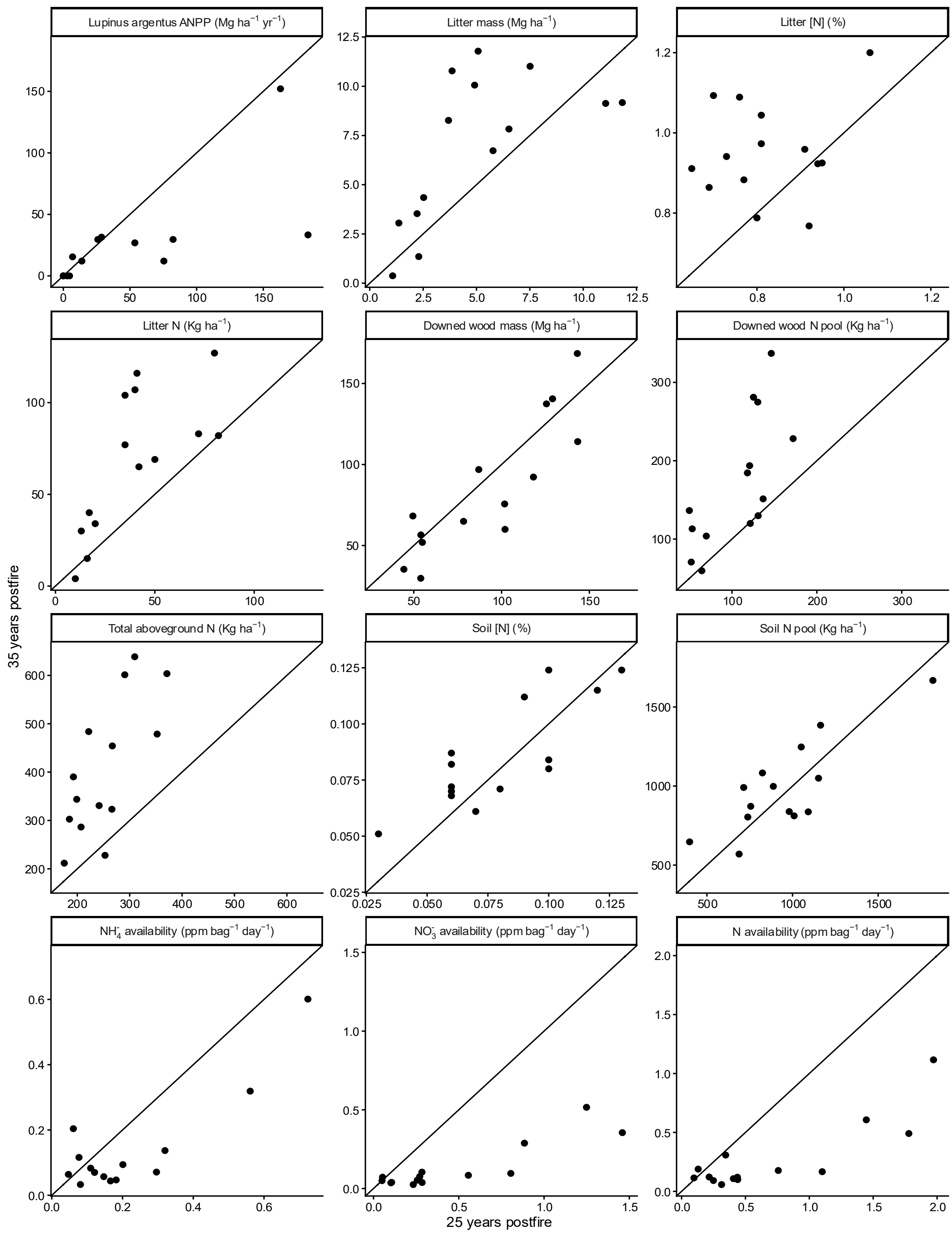

Figure S1: 1:1 plots of lodgepole pine density and productivity as well as ecosystem N pools, concentrations, and availability at 25 and 35 years postfire. Note that x and y axes values differ among figure panels and units are included in panel labels.

Table S2. Correlations of N pools, concentrations, and availability with lodgepole pine ANPP, stem density, and aboveground biomass at 25 and 35 years postfire. Values are Pearson correlation coefficients with *p* values in brackets (*n* = 14 for all analyses). Significant correlations ($p < 0.05$) are in bold text.

**Lodgepole pine:**	**ANPP (Mg ha^{-1} yr^{-1})**		**Stem density (log_{10} stems ha^{-1})**		**Aboveground biomass (Mg ha^{-1})**	
**Years postfire:**	**25**	**35**	**25**	**35**	**25**	**35**
**Variable:**						
ANPP (Mg ha^{-1} yr^{-1})	*NA*	*NA*	0.66 [0.01]	0.91 [< 0.001]	**0.99 [< 0.001]**	**0.77 [0.001]**
Stem density (stems ha^{-1}; log_{10})	**0.66 [0.01]**	**0.91 [< 0.001]**	*NA*	*NA*	**0.55 [0.043]**	0.46 [0.10]
Aboveground biomass (Mg ha^{-1})	**0.99 [< 0.001]**	**0.77 [0.001]**	**0.55 [0.04]**	0.46 [0.10]	*NA*	*NA*
Foliar N (%)	**-0.82 [< 0.001]**	**-0.55 [0.04]**	**-0.83 [< 0.001]**	-0.48 [0.08]	**-0.76 [0.002]**	-0.44 [0.12]
Foliar N pool (kg N ha^{-1})	**0.92 [< 0.001]**	**0.79 [0.001]**	0.35 [0.22]	0.53 [0.05]	**0.97 [< 0.001]**	**0.96 [< 0.001]**
Total tree N pool (kg N ha^{-1})	**0.93 [< 0.001]**	**0.77 [0.001]**	0.35 [0.22]	0.5 [0.07]	**0.97 [< 0.001]**	**0.97 [< 0.001]**
Downed wood N pool (kg N ha^{-1})	**-0.64 [0.01]**	-0.47 [0.09]	**-0.66 [0.01]**	-0.34 [0.23]	**-0.57 [0.03]**	-0.38 [0.19]
Litter N (%)	-0.51 [0.06]	0.14 [0.62]	-0.48 [0.09]	0.09 [0.76]	-0.47 [0.09]	0.29 [0.31]
Litter N pool (kg N ha^{-1})	**0.6 [0.02]**	0.53 [0.05]	**0.69 [0.01]**	0.47 [0.09]	0.52 [0.06]	**0.58 [0.03]**
Total aboveground N (kg N ha^{-1})	**0.70 [0.01]**	0.43 [0.15]	0.20 [0.50]	0.33 [0.27]	**0.76 [0.002]**	**0.62 [0.02]**
Soil N (%)	-0.32 [0.26]	-0.16 [0.59]	-0.18 [0.53]	-0.37 [0.19]	-0.33 [0.25]	0.23 [0.43]
Soil N pool (kg N ha^{-1})	-0.38 [0.18]	-0.34 [0.24]	-0.39 [0.17]	**-0.56 [0.04]**	-0.35 [0.22]	0.15 [0.60]
Resin-sorbed NH_4^+ (ppm bag^{-1} day^{-1}; log_{10})	**-0.6 [0.02]**	-0.35 [0.23]	**-0.59 [0.03]**	-0.31 [0.23]	**-0.57 [0.04]**	-0.25 [0.39]
Resin-sorbed NO_3^- (ppm bag^{-1} day^{-1}; log_{10})	-0.39 [0.16]	**-0.55 [0.04]**	**-0.69 [0.01]**	-0.53 [0.05]	-0.31 [0.27]	-0.36 [0.20]
Total resin-sorbed N (ppm bag^{-1} day^{-1}; log_{10})	-0.47 [0.09]	-0.46 [0.10]	**-0.68 [0.01]**	-0.43 [0.13]	-0.41 [0.15]	-0.31 [0.28]

Table S3. Results of Pearson correlation analysis of potential N-fixers with lodgepole pine foliar N pools and concentration. Additionally, relationships of soil N pools and percent clay as well as soil C and N concentrations are shown. Significant correlations ($p < 0.05$) are in bold text ($n = 14$ for all analyses). All correlations are for 35-years postfire.

**Variable 1**	**Variable 2**	**Pearson's *r***	***p***
Silvery lupine ANPP (Kg ha^{-1} $year^{-1}$) [Log_{10} transformed]	Lodgepole pine foliar N pool (Kg N ha^{-1})	-0.28	0.34
	Lodgepole pine foliar [N] (%)	**0.55**	**0.04**
Moss % cover	Lodgepole pine foliar N pool (Kg N ha^{-1})	-0.42	0.13
	Lodgepole pine foliar [N] (%)	**0.66**	**0.01**
Lichen % cover [Log_{10} transformed]	Lodgepole pine foliar N pool (Kg N ha^{-1})	0.84	0.06
	Lodgepole pine foliar [N] (%)	0.24	0.40
Soil [N] (%)	Soil [C] (%)	**0.98**	**<0.001**
Soil N pool (Kg ha^{-1})	Soil clay (%)	**0.60**	**0.02**

Table S4. Correlations (Pearson) among N pools for 25 and 35 years postfire as well as annual rates of change in N pools from 25-35 years postfire. Significant correlations ($p < 0.05$) are in bold text ($n = 14$ for all analyses).

		**2012**		**2022**		**Annual rate of change (Δ)**	
**N pools 1 (Kg ha^{-1})**		Pearson's *r*	*p*	Pearson's *r*	*p*	Pearson's *r*	*p*
Aboveground lodgepole pine	Litter	0.41	0.14	**0.62**	**0.02**	0.45	0.10
	Downed wood	-0.45	0.10	-0.26	0.38	-0.03	0.92
	Soil	-0.26	0.36	0.03	0.93	0.13	0.66
Litter	Downed wood	-0.45	0.11	0.14	0.63	0.16	0.59
	Soil	-0.33	0.25	-0.33	0.26	0.12	0.67
Downed wood	Soil	**0.55**	**0.04**	-0.06	0.85	-0.42	0.13
Total aboveground	Soil	-0.03	0.93	-0.10	0.73	-0.14	0.64

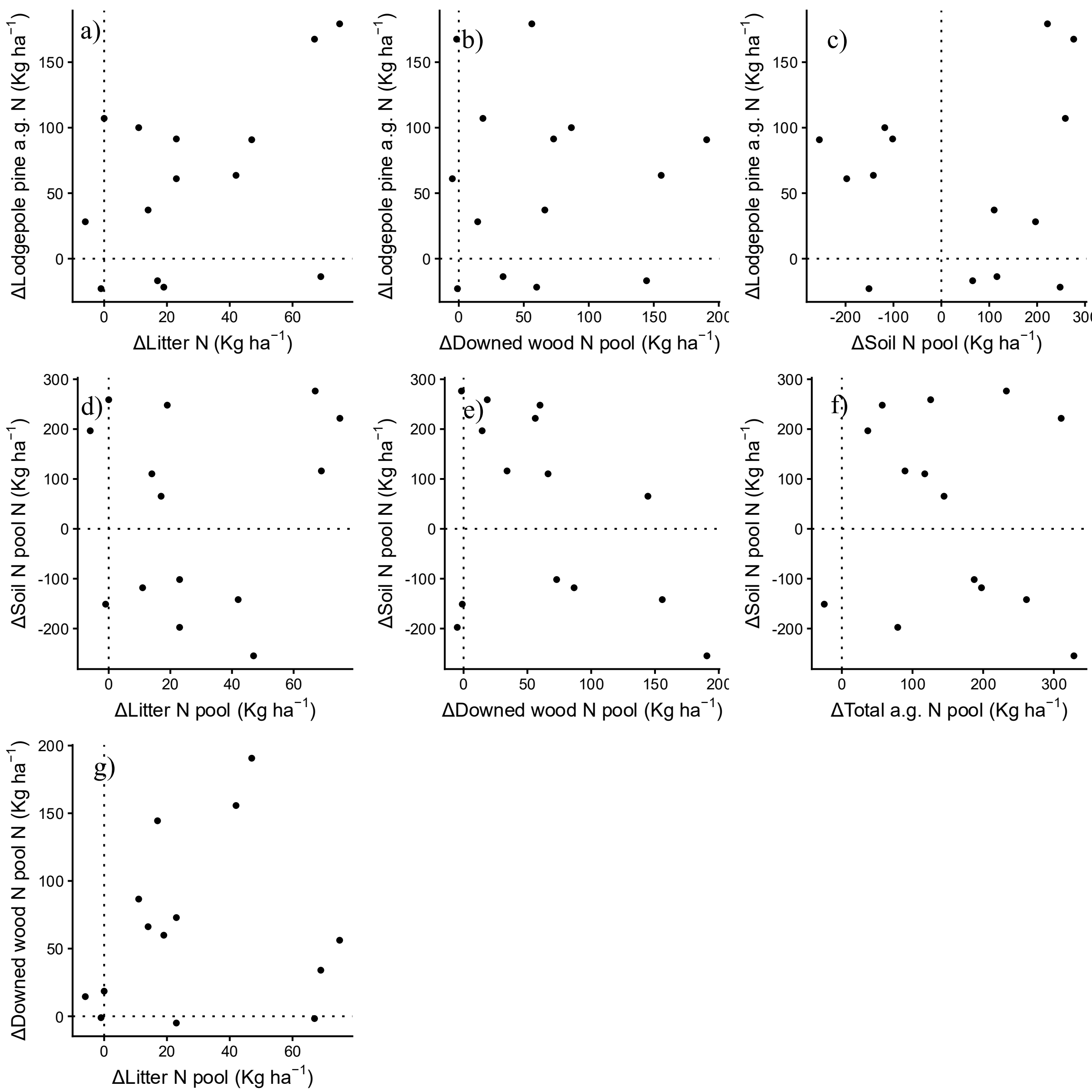

Figure S2: Scatterplots of decadal rates change in N pools from 25 to 35 years postfire. No correlations (Pearson's) were significant (all $p > 0.05$; $n = 14$ for all analyses). The abbreviation "a.g." stands for "aboveground".

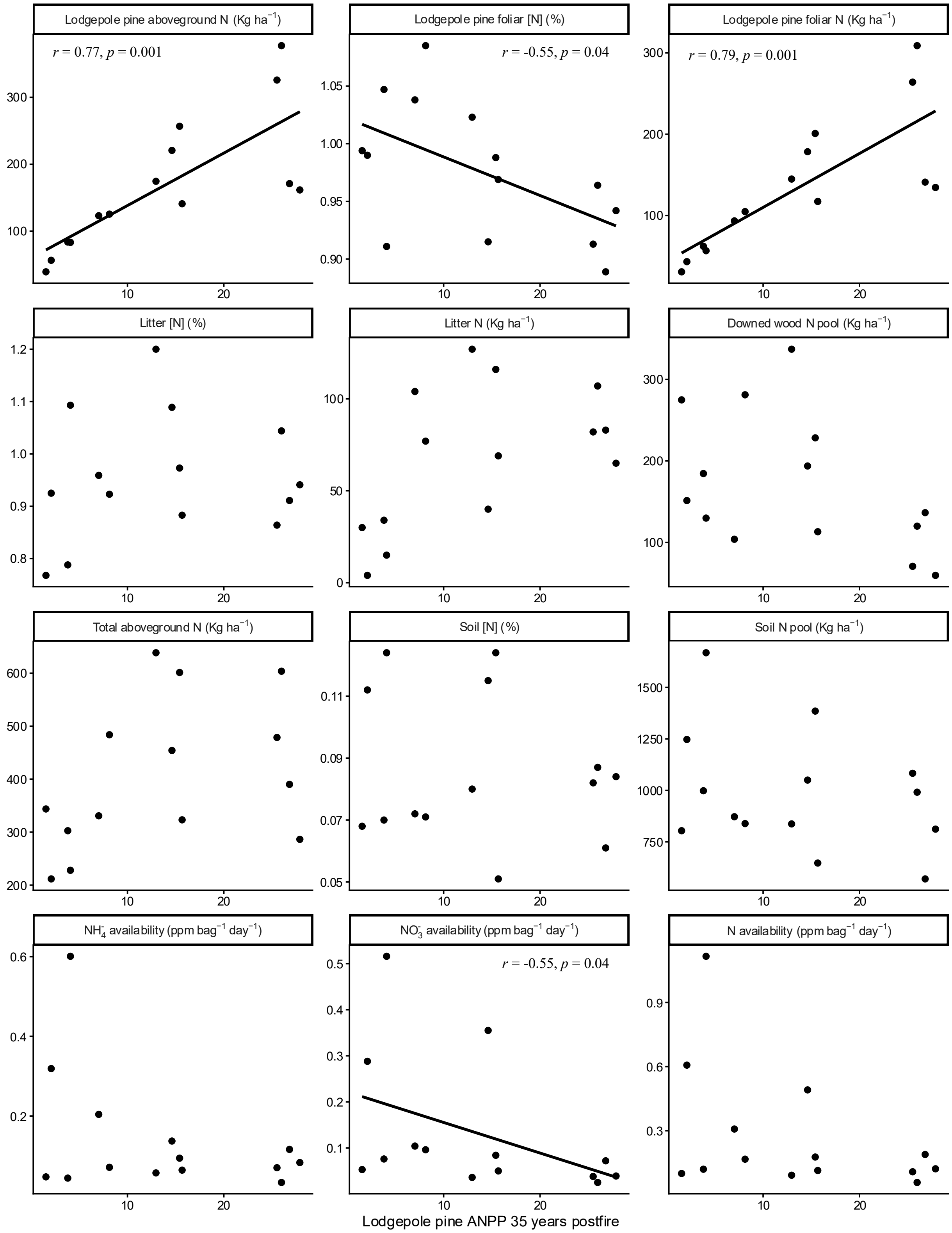

Figure S3: Scatter plot showing different N cycle metrics (Y-axis, with variables noted on individual panel labels) versus lodgepole pine ANPP (Mg ha^{-1} year^{-1}) 35-years postfire (X-axis). Trendlines depict significant Pearson correlations ($p < 0.05$; $n = 14$ for all analyses).

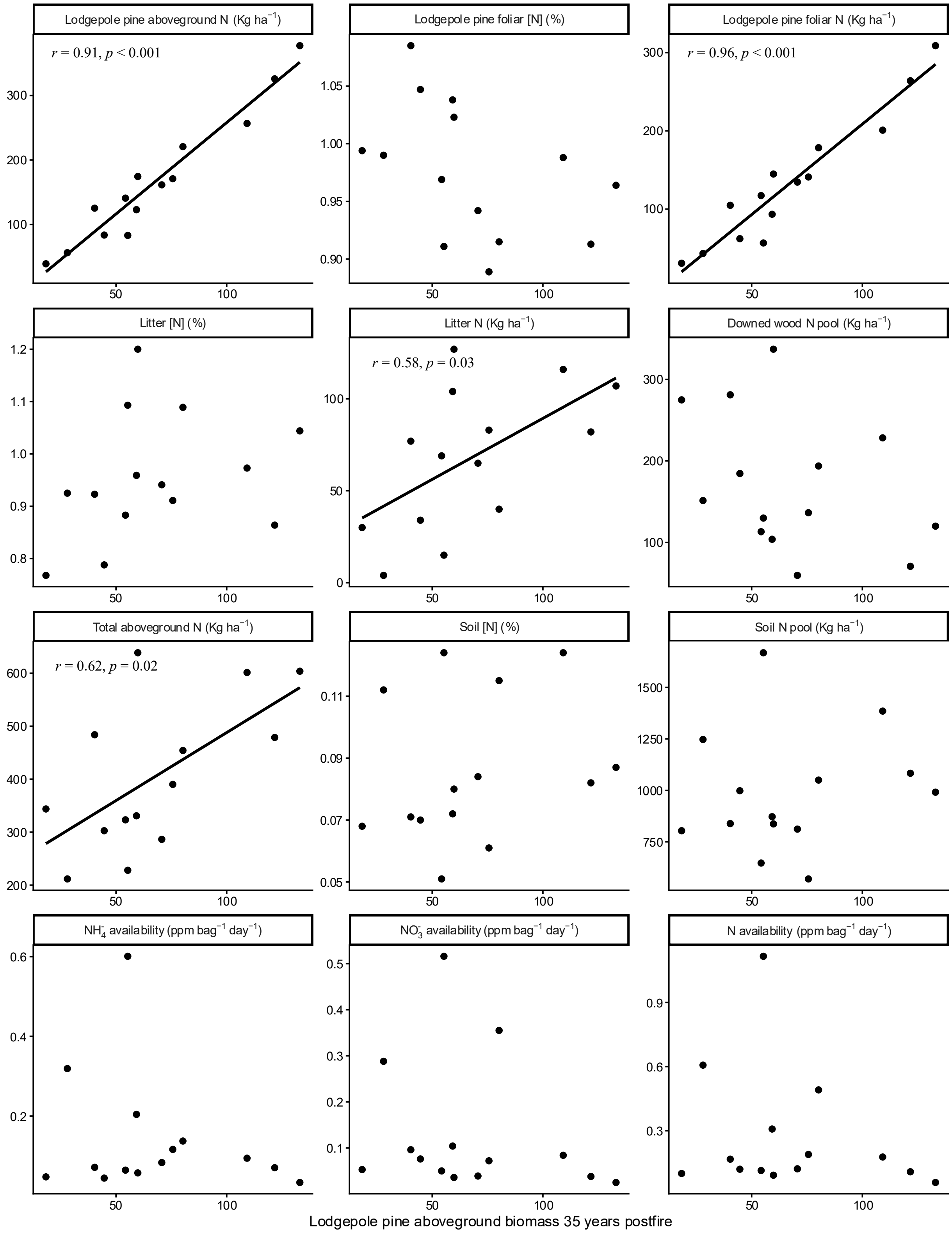

Figure S4: Scatter plot showing different N cycle metrics (Y-axis, with variables noted on individual panel labels) versus lodgepole pine biomass (Mg ha^{-1}) 35-years postfire (X-axis). Trendlines depict significant Pearson correlations ($p < 0.05$; $n = 14$ for all analyses).

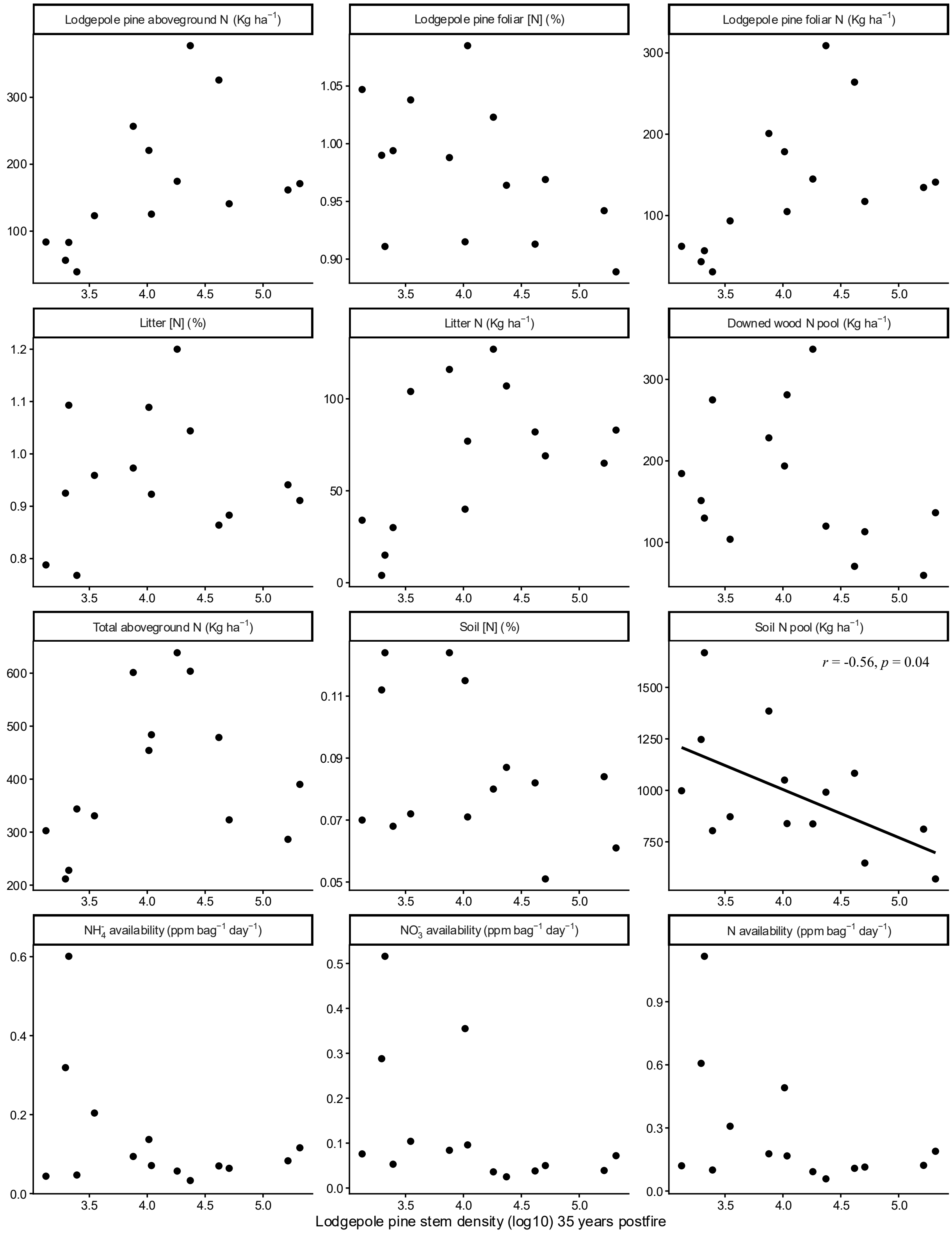

Figure S5: Scatter plots of N cycle metrics (Y-axis, with variables noted on individual panel labels) versus lodgepole pine stem density (n ha^{-1}, $\log_{10}$ transformed) 35-years postfire (X-axis). Trendlines depict significant Pearson correlations ($p < 0.05$; n = 14 for all analyses).

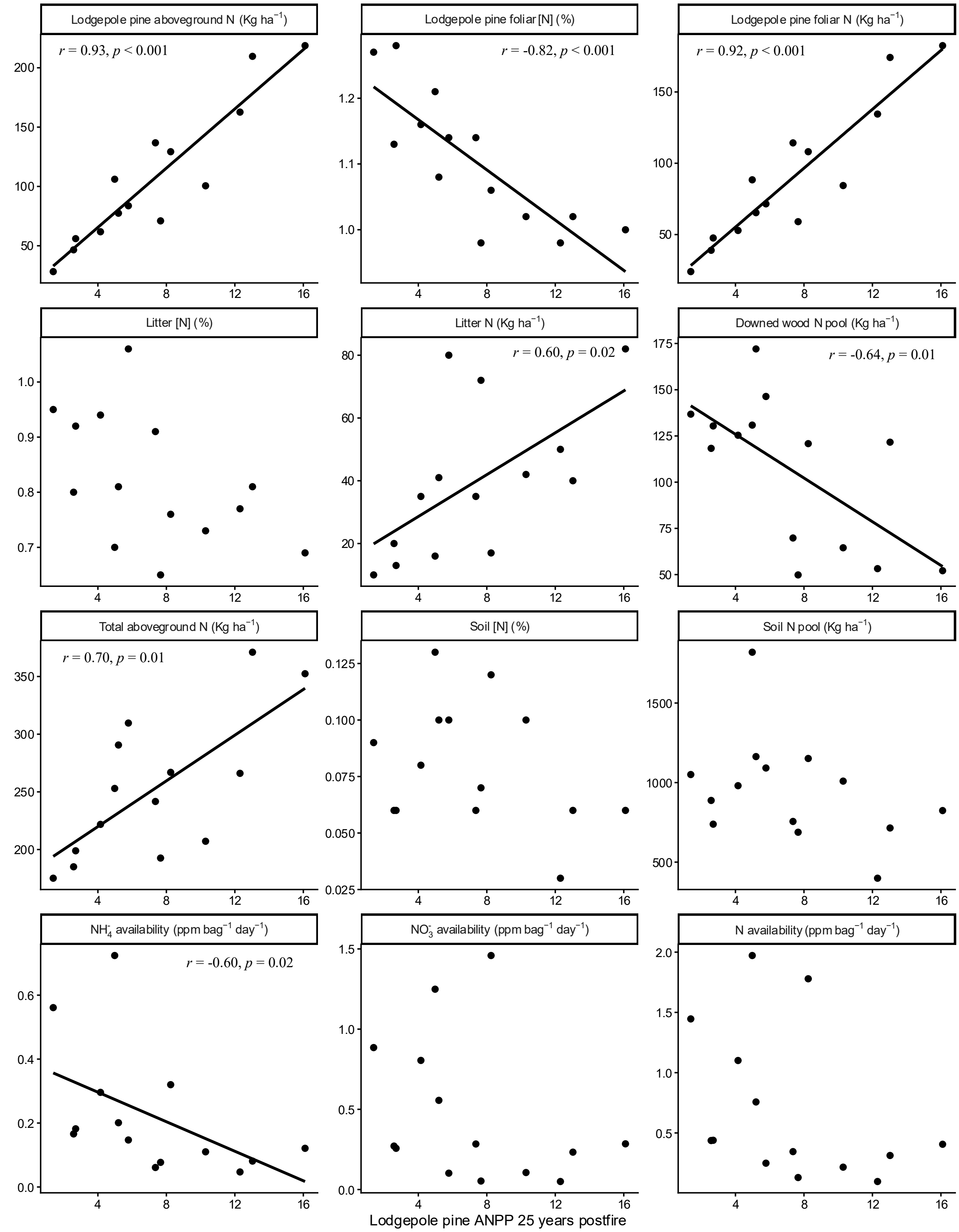

Figure S6: Scatter plots of N cycle metrics (Y-axis, with variables noted on individual panel labels) versus lodgepole pine ANPP (Mg ha^{-1} year^{-1}) 25-years postfire (X-axis). Trendlines depict significant Pearson correlations ($p < 0.05$; $n = 14$ for all analyses).

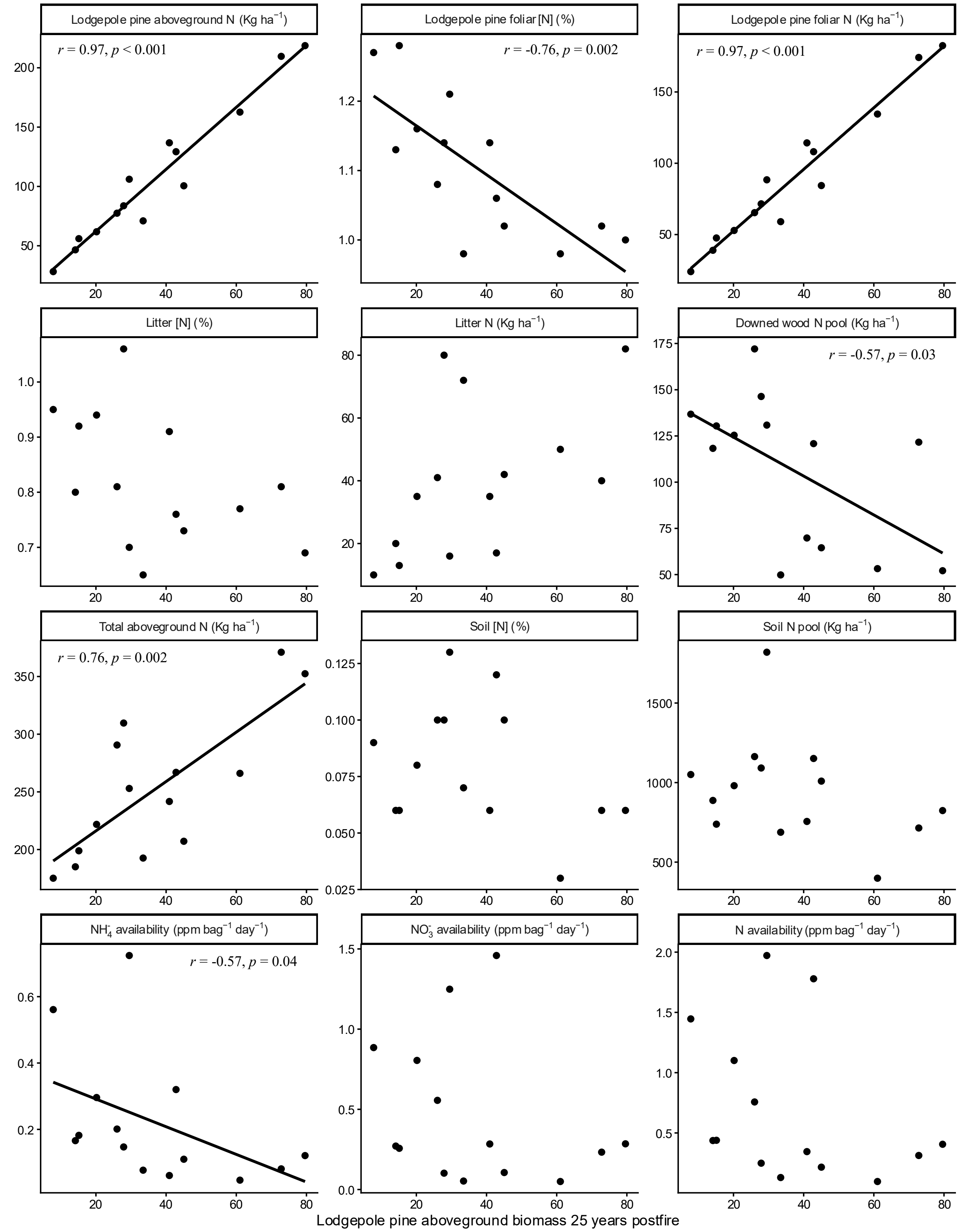

Figure S7: Scatter plots of N cycle metrics (Y-axis, with variables noted on individual panel labels) versus lodgepole pine biomass (Mg ha^{-1}) 25-years postfire (X-axis). Trendlines depict significant Pearson correlations ($p < 0.05$; $n = 14$ for all analyses). .

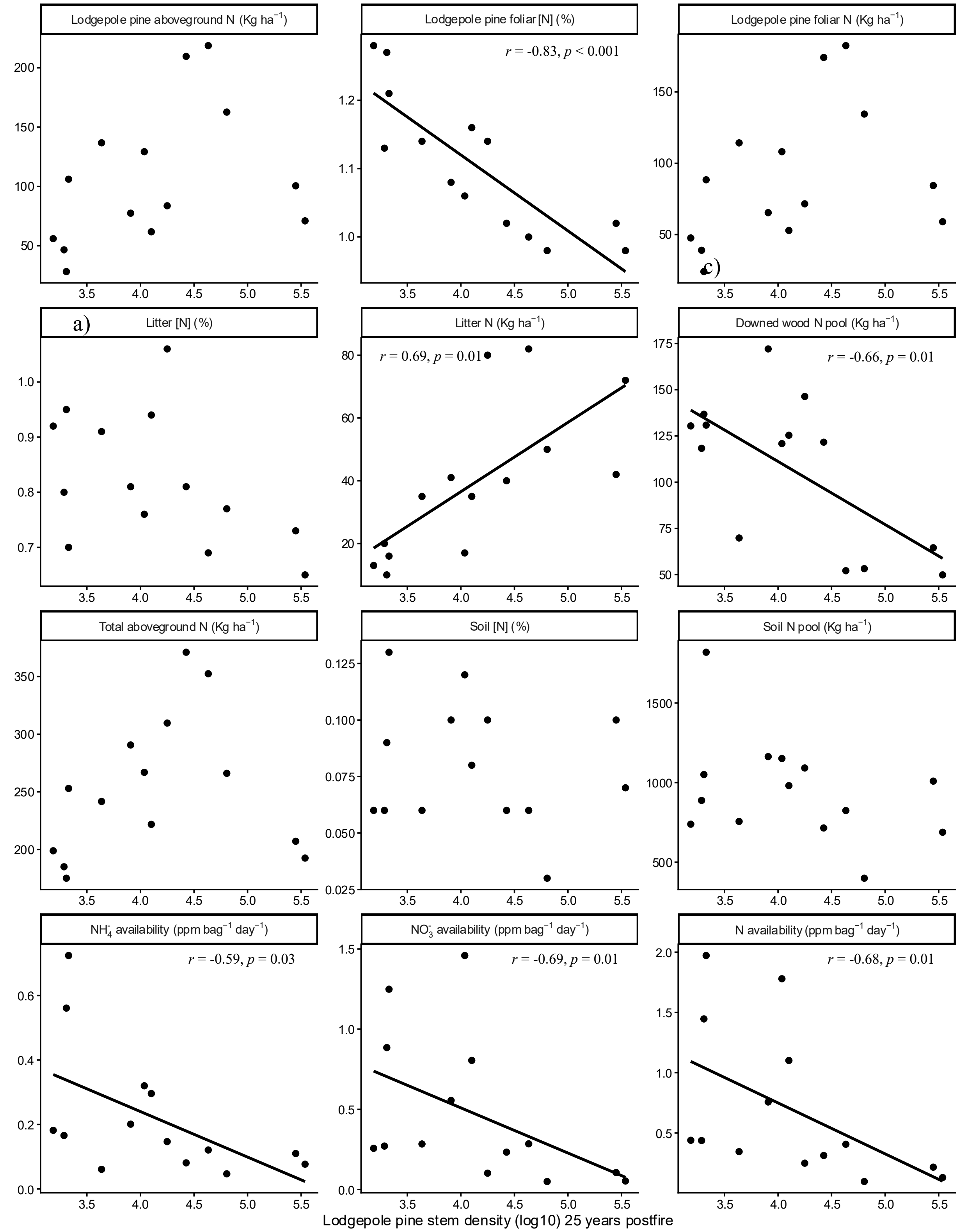

Figure S8: Scatter plots of N cycle metrics (Y-axis, with variables noted on individual panel labels) versus lodgepole pine stem density (n ha^{-1}, $\log_{10}$ transformed) 25-years postfire (X-axis). Trendlines depict significant Pearson correlations ($p < 0.05$; $n = 14$ for all analyses).

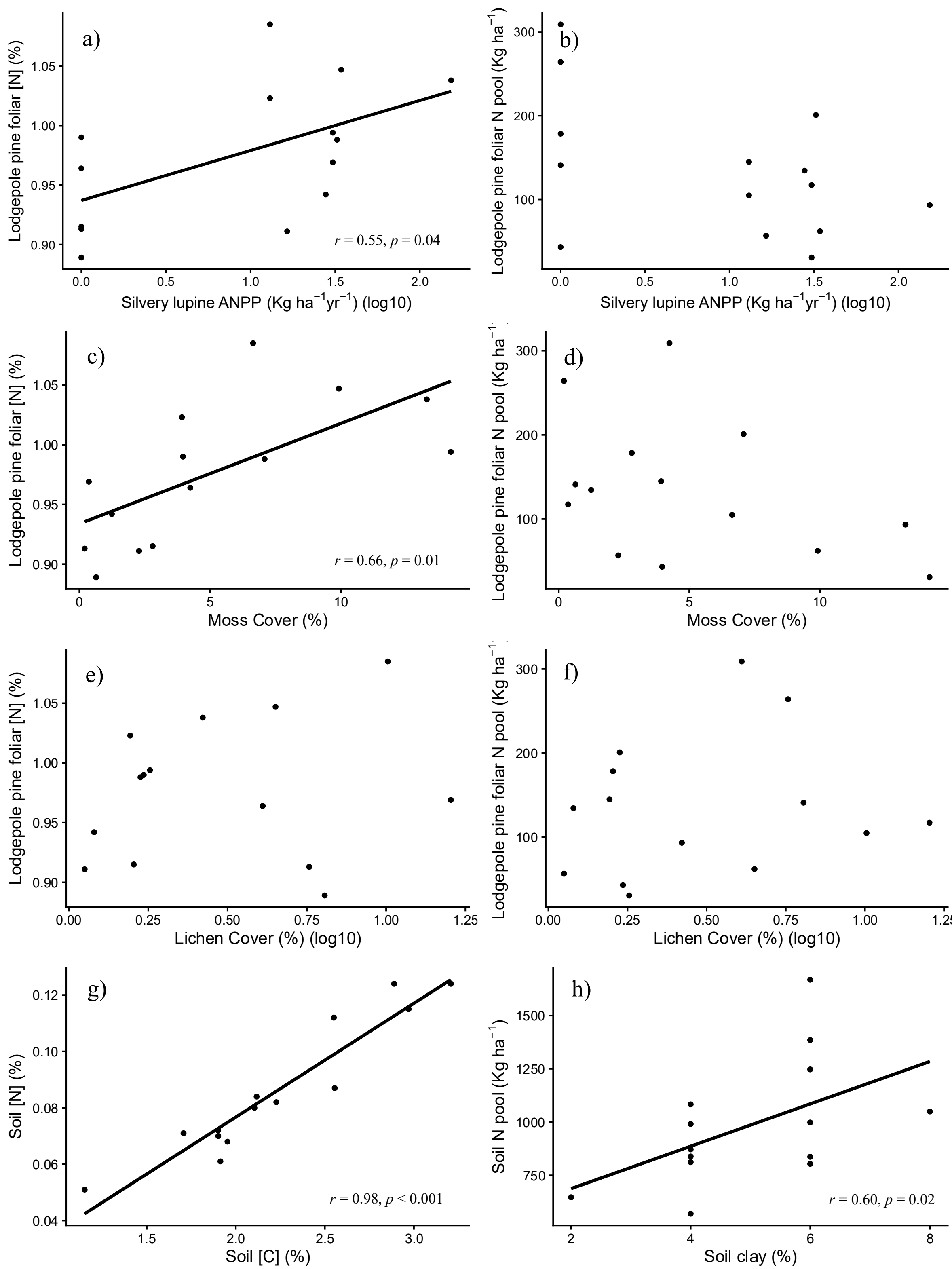

Figure S9: Scatterplots of lodgepole pine foliar N pools and concentrations versus potential N fixers as well as soil C and N concentrations and soil N pools versus soil percent clay. Scatterplots among N pools at 35 years postfire. Trendlines depict significant Pearson correlations ($p < 0.05$; n = 14 for all analyses). "A.g." stands for "aboveground".

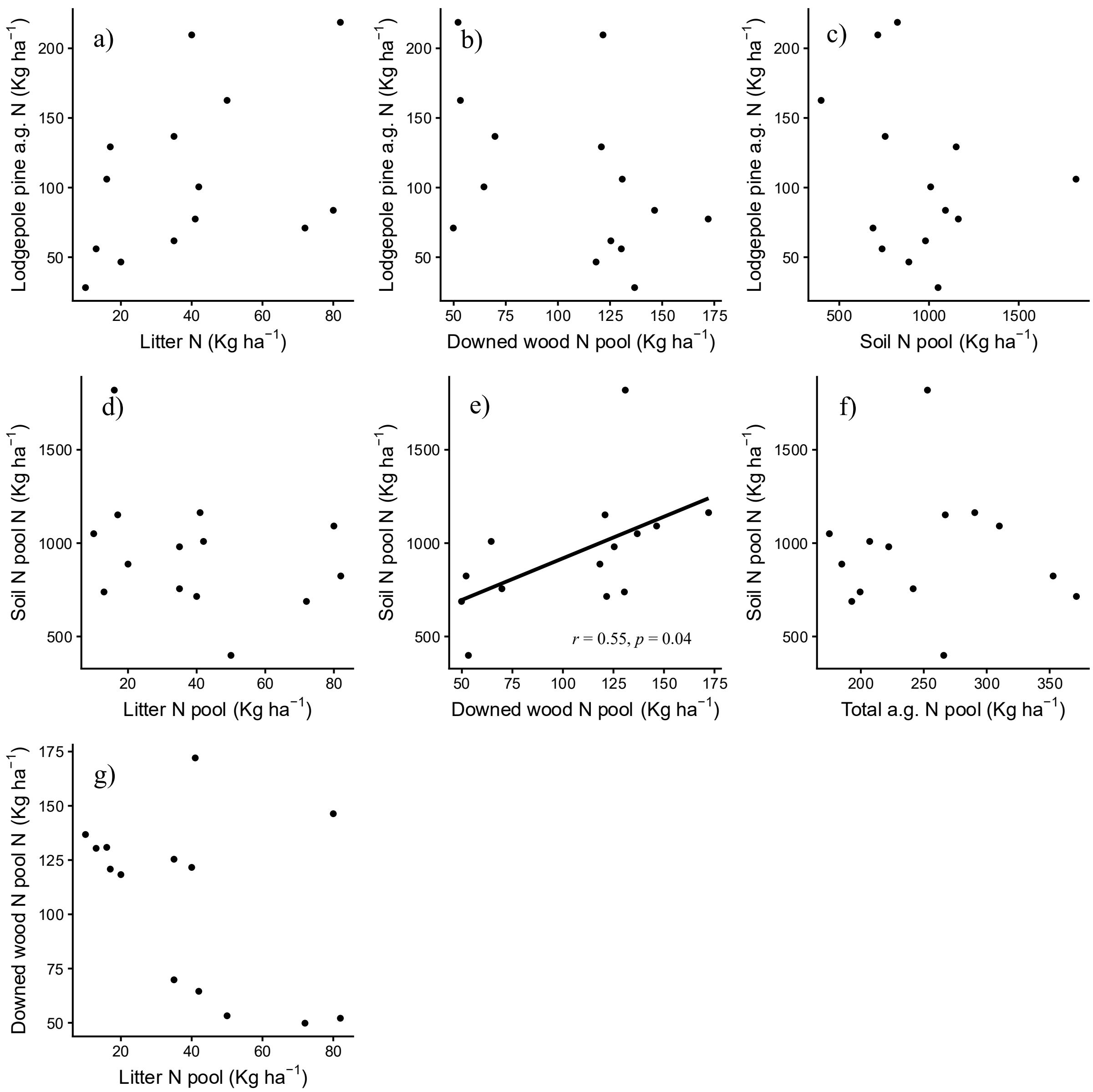

Figure S10: Scatterplots among N pools at 25 years postfire. Trendlines depict significant Pearson correlations ($p < 0.05$; $n = 14$ for all analyses). "A.g." stands for "aboveground".

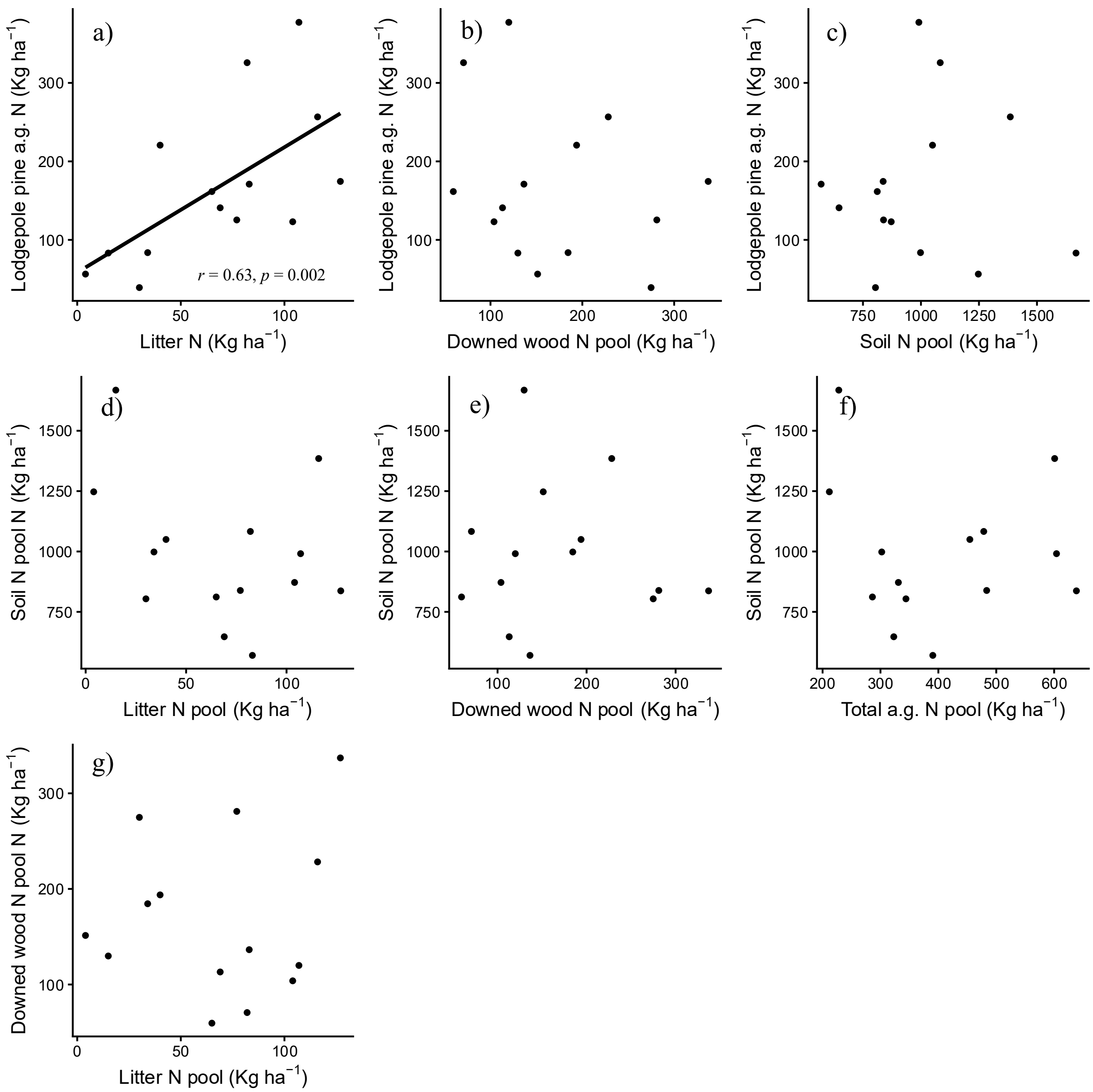

Figure S11: Scatterplots among N pools at 35 years postfire. Trendlines depict significant Pearson correlations ($p < 0.05$). The abbreviation "a.g." stands for "aboveground".
