# Supplementary material for: Nitrogen dynamics 35 years after stand‐replacing fire: Aboveground pools grow as surface soil pools remain unchanged
Source: Ecology. 2026 Oct 6;107(10):e70546. doi: 10.1002/ecy.70546 (PMC13640499; doi:10.1002/ecy.70546)
Supplement: Supplementary file 2 [file ECY-107-e70546-s002.pdf]

Appendix S2: Supplemental analyses

Nitrogen dynamics 35 years after stand-replacing fire: Aboveground pools grow as surface soil pools remain unchanged

Timon T. Keller, Cory C. Cleveland, Robert E. Heumann, Arielle C. Link, Lucille T. McGuire, and Monica G. Turner

Ecology

Section S1. Calculating aboveground net primary productivity at 35-years postfire

Overview

To estimate aboveground net primary productivity (ANPP) of lodgepole pine (*Pinus contorta* var. *latifolia*), we used allometric equations developed by Copenhaver and Tinker (2014). Out of the 350 trees we measured across 14 plots, 22 trees exceeded the maximum basal diameter of Copenhaver and Tinker's sample. Alternative methods to calculate ANPP for larger lodgepole pines require radial increment at DBH and tree height or height from base to live crown (for example, Comeau and Kimmins 1989, Litton et al. 2004, Kashian et al. 2013). While lodgepole pine height can be estimated from allometric equations (e.g. Turner et al. 2025), we did not measure radial increment in the field and could not use those methods to estimate ANPP for large lodgepole pines. Therefore, we evaluated the consequences of three options for computing ANPP at the plot level.

Methods

Since only a small proportion of our trees exceeded the maximum tree sizes used to develop the allometric equations, we tested three methods for estimating ANPP using equations from Copenhaver and Tinker (2014). For method 1, we used equations from Copenhaver and Tinker (2014) for the 22 trees that are too large for the equations. For method 2, we reduced the measured basal diameters of the 22 large trees to the maximum basal diameter in the Copenhaver and Tinker dataset for trees that were sampled destructively. For method 3, we removed the 22 large trees from our dataset and recalculated ANPP only for trees that were within range of Copenhaver and Tinker's equations. For each of the three methods, we calculated mean ANPP ha^{-1} by transect (i.e., computing ANPP for each measured tree, calculating mean ANPP per transect, then multiplying by tree density on that transect), averaged at the plot level, and then averaged across all plots.

As lodgepole pines mature, ANPP peaks around 40-70 years postfire (Kashian et al. 2013). The Copenhaver and Tinker (2014) allometric equations model an exponential increase in ANPP with basal diameter and perform well across the tree sizes in their sample. Thus, we expected that method 1 would overestimate ANPP if growth of the larger trees was beginning to slow down. We expected that method 2 would slightly underestimate ANPP because we reduced the maximum ANPP of the larger trees. We expected that method 3, in which large trees were removed, would underestimate ANPP because highly productive individuals were omitted.

To assess the sensitivity of our results to these methods, we re-calculated means and standard errors of lodgepole pine ANPP across the 14 plots. Because large trees were distributed among plots and transects, and ANPP of 8 or 9 trees was averaged by transect before plot-level estimates were obtained, we expected that differences among methods could be small.

Results

Means and standard errors for lodgepole pine ANPP did not differ among the three methods (Figure S1). At 35 years postfire, lodgepole pine ANPP across our plots remained strongly driven by smaller trees within range of Copenhaver and Tinker's allometric equations and by the variation in tree density among the plots. How ANPP was calculated for the 22 largest trees had no effect on the final estimates of ANPP at the plot level. We selected the "middle of the road" approach (method 2) that reduced measured DBH to the maximum DBH in the Copenhaver and Tinker (2014) dataset to estimate ANPP.

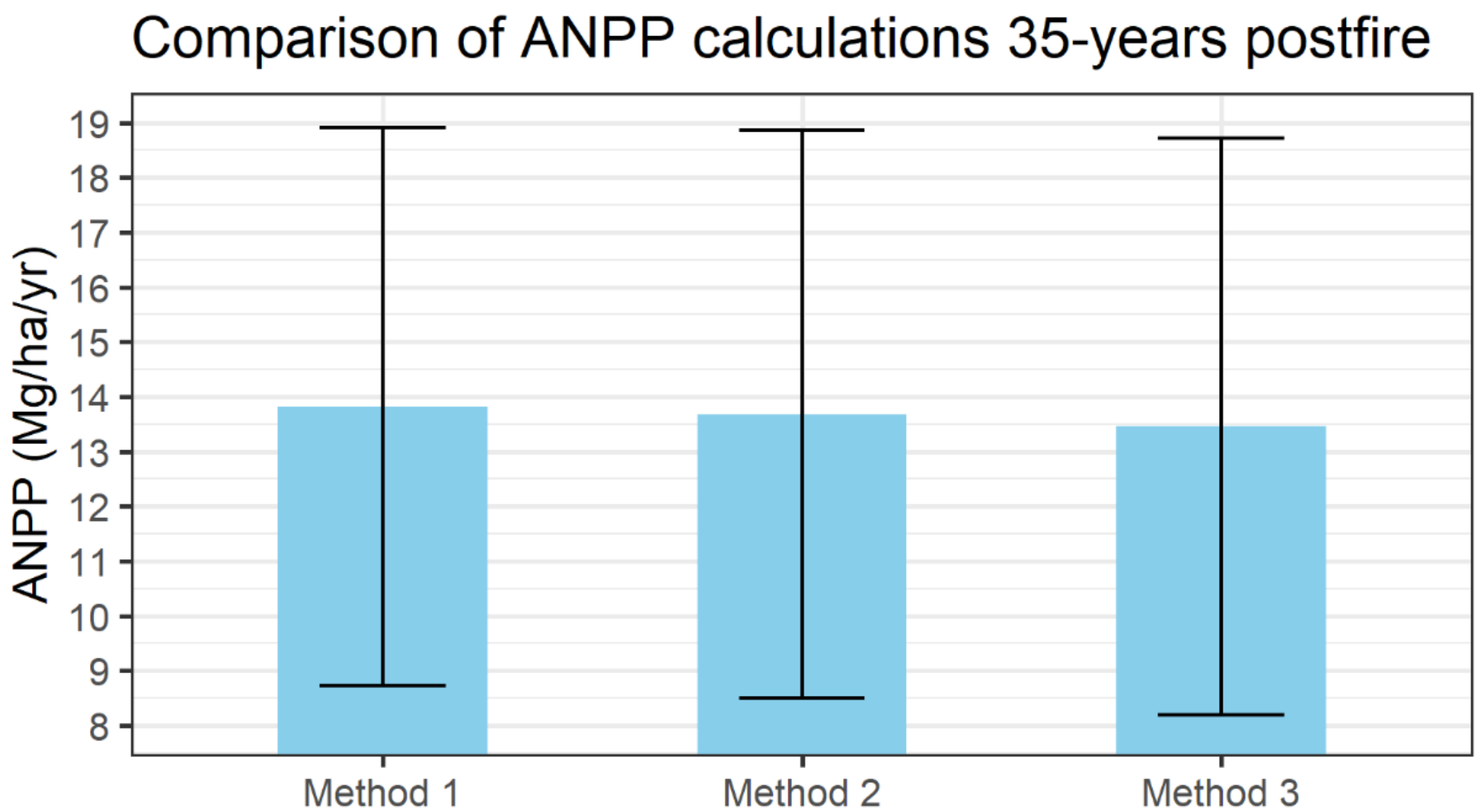

Figure S1: Mean and ± 2 SE of ANPP (MG ha^{-1} $year^{-1}$) calculated using the three different methods described above. Method 1 applied the same allometric equations (Copenhaver and Tinker 2014) for all trees sampled. Method 2 reduced the basal diameter of the largest trees to the maximum basal diameter used to developed the allometrics for 25-yr old lodgepole pines. Method 3 eliminated from the dataset the 22 trees that were larger than the sample used to develop the allometric equations.

Section S2. Estimating soil N pools 15 and 25 years postfire

Turner et al. (2019) did not measure soil fine fraction bulk density (hereafter: bulk density) 15 or 25 years postfire, assuming no change over time or variation among stands in bulk density. Instead, Turner et al. (2019) calculated soil N pools using a bulk density estimate of 1 g cm^{-3}, which represents the mean from the top 15 cm of soil in 17 plots of 12–35 year old postfire lodgepole pine in Yellowstone National Park (Smithwick et al. 2009, Kashian et al. 2013). However, bulk density decreases between 10 – 40 years postfire (Figure 8c in Smithwick et al. 2009), and a mean value calculated from 12 – 35 year old stands may not accurately represent bulk density at 15 or 25 years postfire. Further, a mean value does not capture variation in bulk density across the landscape (i.e., among stands).

To reconcile this, we modeled bulk density values for 15 and 25-year-old stands and recalculated soil N pools using soil N concentrations measured in the field at both times postfire. For our model of soil bulk density, we used data from a lodgepole pine chronosequence in Yellowstone National Park (Smithwick et al. 2009), an ongoing N fertilization experiment in Grand Teton National Park (Turner et al., unpublished data), and this study. We only included bulk density measurements from stands less than 100 years old to better constrain the model.

We used segmented regression (Toms and Lesperance 2003) to model changes in bulk density over time because visual inspection of the data (Figure S2) suggested a non-linear association between stand age and bulk density in stands younger than ~40 years. We also considered a logarithmic model (bulk density predicted by the natural log of stand age; $R^2 = 0.45$, $\beta = -0.15$, SE = 0.02, $p < 0.001$, AIC = -68.7) and a negative polynomial model (bulk density predicted by a second order polynomial function; $R^2 = 0.49$, $\beta_1 = -0.63$, $\beta_2 = 0.55$, SE = 0.12, $p < 0.001$, AIC = -71.5). However, the segmented regression model (bulk density predicted by natural log of stand age) fit the data best ($R^2 = 0.53$, $p = 0.001$, AIC = -75.1), and we used this model to estimate soil bulk density at 15 and 25 years postfire.

Rather than using our final model to predict a mean bulk density value at 15 and 25 years postfire, we used the model to "backcast" bulk density for each plot based on measured bulk density 35 years postfire. This approach retains the variation in bulk density observed among the plots at 35 years postfire and captures the change in bulk density over time. Further, this approach aligns with statistical assumptions of linear regression because residuals are expected to be normally distributed along the range of predictor values (here, stand age). Thus, the model does not assume that bulk density converges over time as stands age.

The segmented regression model estimated a breakpoint at 28.5 years, and only the slope coefficient for the model from 0 – 28.5 years was significantly different from zero ($\beta_{<28.5yrs} = -0.25$, $SE_{<28.5yrs} = 0.06$, $p = 0.001$; $\beta_{>28.5yrs} = 0.01$, $SE_{>28.5yrs} = 0.06$, $p = 0.87$). Thus, we reasoned that bulk density at 28.5 and 35 years postfire were not different and used measured bulk density values from 35-years postfire to backcast beginning from 28.5 years postfire. We backcast every plot location individually by adjusting the model intercept by the difference between the observed value and the mean predicted bulk density at 28 years postfire (Figure S3). Lastly, we tested whether these different methods affect our interpretation of no change in soil N pools from 25 – 35 years postfire (Table S1) and visualized trajectories (Figure S4).

Table S1: Comparison of three different methods to calculate soil BD.

**Method**	**Soil N pool at 15 years [mean (SE)]**	**Soil N pool at 25 years [mean (SE)]**	**Soil N pool at 35 years [mean (SE)]**	**Paired t-test 15 – 25 years**	**Paired t-test 25 – 35 years**
Assume bulk density of 1 g cm^{-3} at 15 & 25 years postfire. Assumes constant bulk density among stands.	857 (76)	1,200.0 (111)	985.9 (78.3)	Significant increase ($t = 3.99$, $df = 9$, $p = 0.003$)	Significant decrease ($t = -2.44$, $df = 13$, $p = 0.03$)
Mean bulk density predicted at 15 (0.90 1 g cm^{-3}) & 25 (0.77 1 g cm^{-3}) years postfire. Assumes constant bulk density among stands.	769.5 (92.3)	876.0 (81.2)	985.9 (78.3)	No change over time ($t = 1.43$, $df = 9$, $p = 0.19$)	No change over time ($t = 1.58$, $df = 13$, $p = 0.14$)
Backcast bulk density at 15 & 25 years postfire. Retains variability in bulk density among stands.	800.5 (104.1)	948.1 (87.9)	985.9 (78.3)	Near significant increase ($t = 2.21$, $df = 9$, $p = 0.05$)	No change over time ($t = 0.74$, $df = 13$, $p = 0.47$)

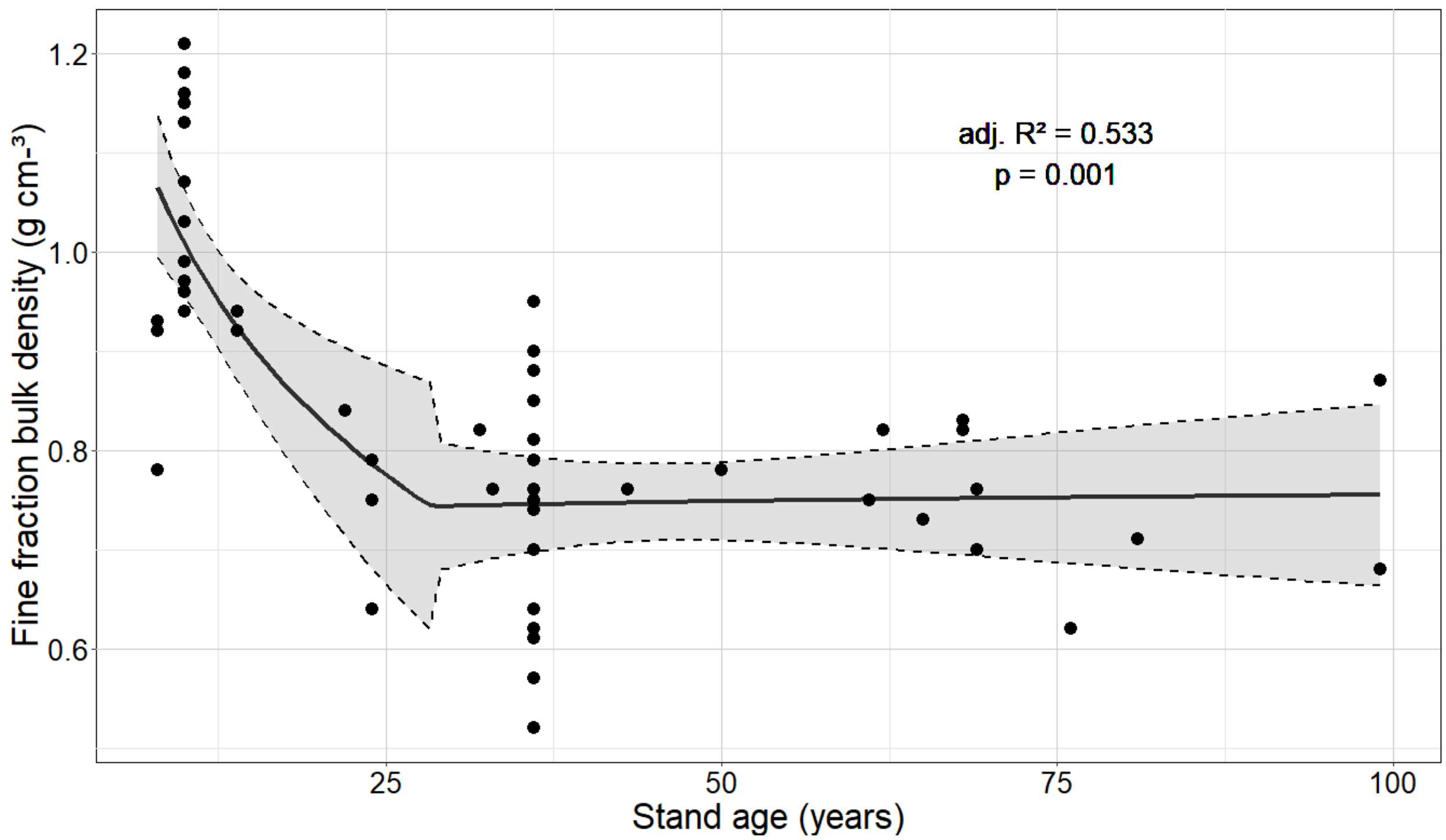

Figure S2: Fragmented regression model fit to fine fraction bulk density and stand age. Shaded area represents 95% confidence intervals. This figure includes data from Smithwick et al. (2009), an ongoing fertilization experiment in Grand Teton (Turner et al., forthcoming), and this study.

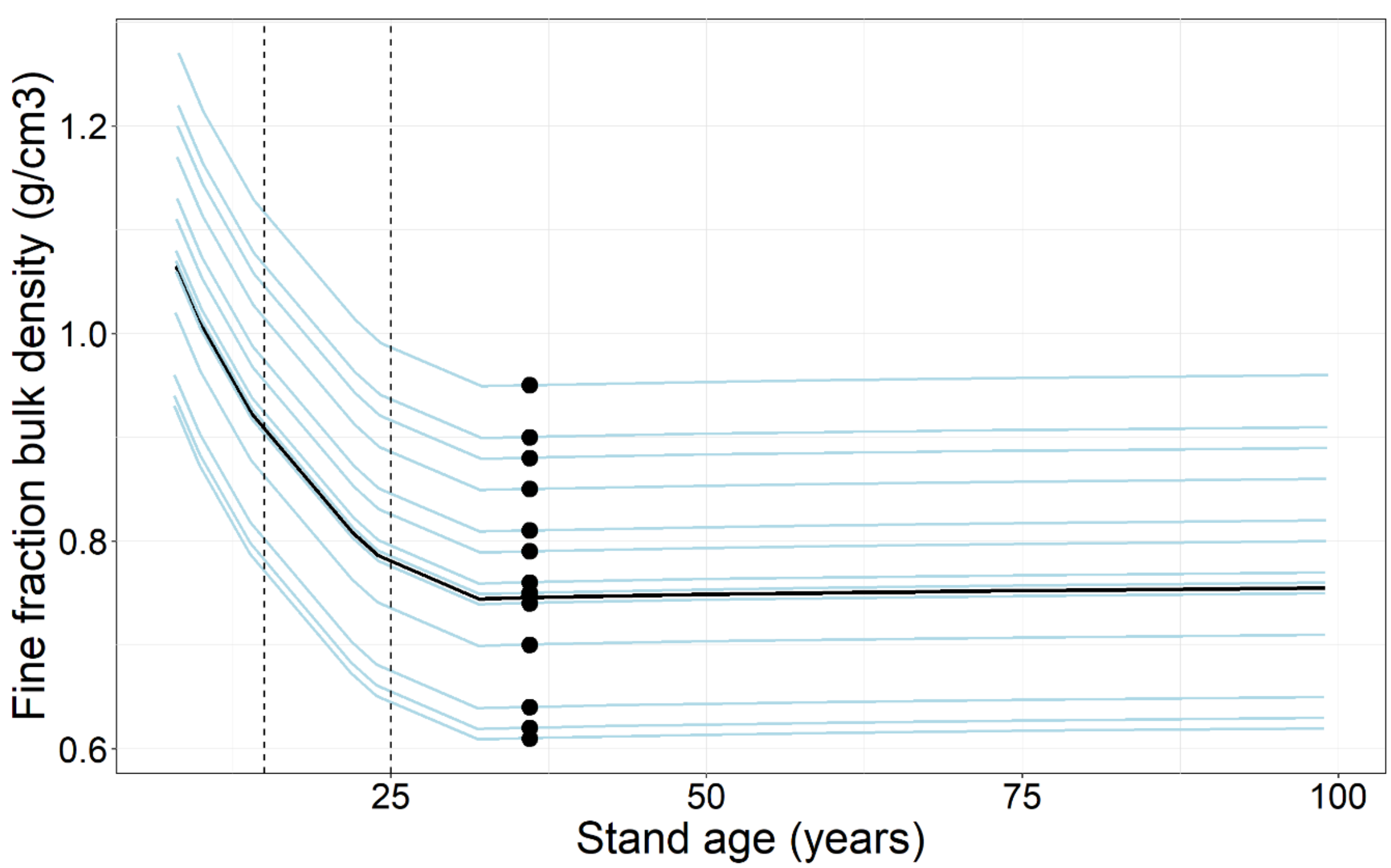

Figure S3: Visualization of adjusting model intercept to backcast soil bulk density for each plot. The black line represents the model equation, and blue lines represent the model with y-intercept adjusted for each measured bulk density at 35-years postfire.

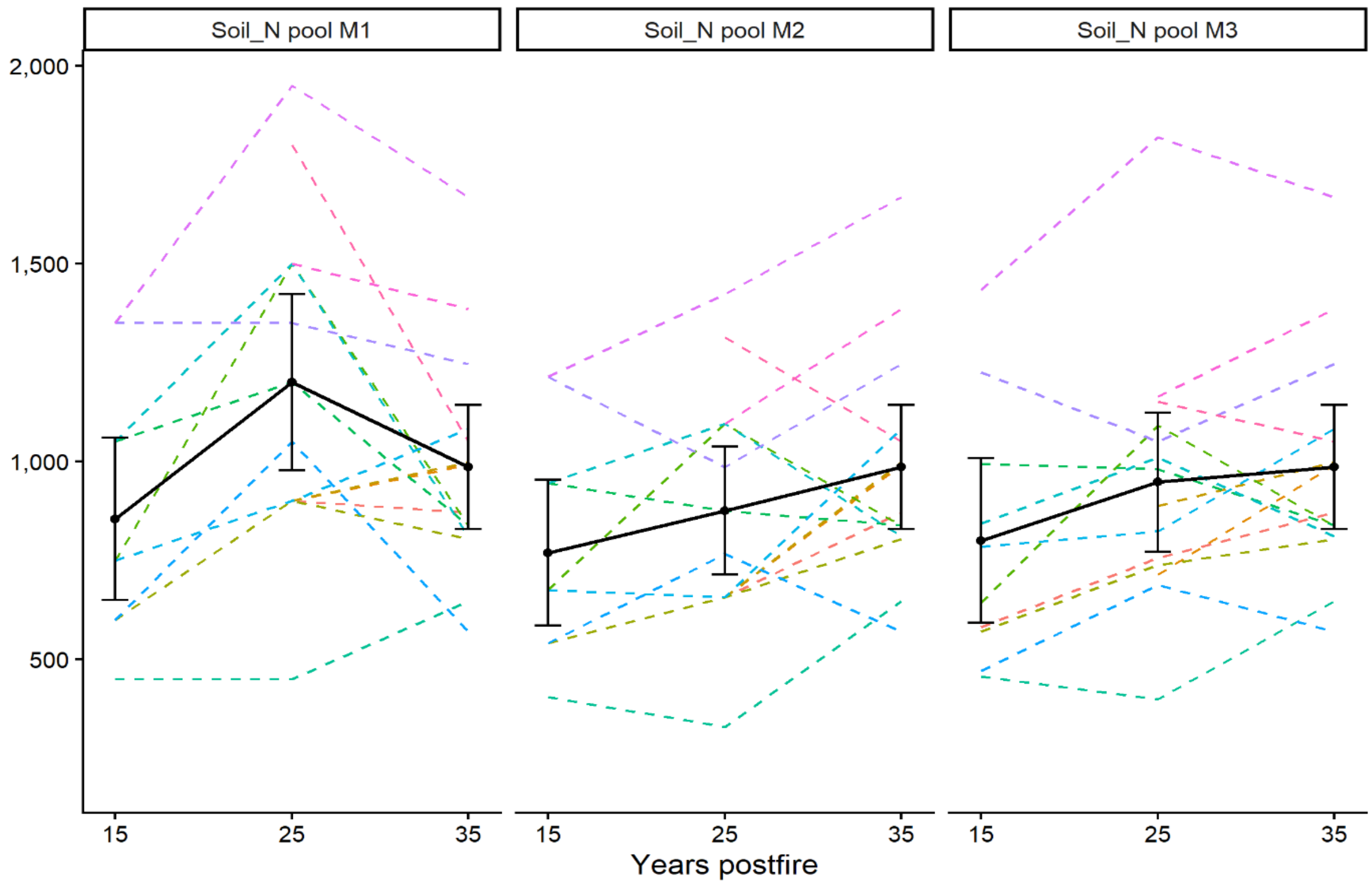

Figure S4: Plot trajectories and mean ± 2SE of soil N pools for the three methods described in Table S1. Note that this includes all 14 plots, thus some lines start at 25 years.

Section S3. Sensitivity analyses of N pool estimates

Overview

Ecosystem nutrient budgets are sensitive to uncertainty in measurements of biomass and nutrient concentrations, but few studies estimate and report such uncertainties (Yanai et al. 2010, 2021, Damgaard 2020). Our study was designed to quantify variation in stem density, aboveground biomass, and ecosystem N pools in aggrading lodgepole pine stands that spanned a wide range of postfire regeneration densities. Here, we estimated sensitivity of our ecosystem N pool estimates to determine whether measurement error could potentially influence our conclusions.

We focused on two key measurements. First, the basal diameter or diameter at breast height (DBH) of measured lodgepole pines, which is used to estimate ANPP and biomass components, and is measured by multiple researchers in the field. Second, N concentrations, which are analyzed on a small mass of homogenized sampled biomass (foliage, litter, soils) or from peer-reviewed literature (downed woody debris). We estimated uncertainty by recalculating N pools assuming ±10% measurement errors of concentration and mass. Further, we also present coefficients of variation (CVs) of N concentrations at the plot level to quantify inherent variation in our samples.

S3.1. Uncertainty in lodgepole pine aboveground biomass & N pools

Approach

We estimated lodgepole pine aboveground biomass using empirical tree measurements (n = 25 trees plot^{-1}) and non-linear allometric equations that use either tree DBH (Pearson et al. 1984) or tree basal diameter (BD, Copenhaver and Tinker 2014). The non-linearity of the allometric equations means measurement errors of DBH or BD may compound exponentially. Thus, we assessed uncertainty by recalculating lodgepole pine N pools with varying (1) estimates of DBH or BD alone by ± 10%, (2) estimates of N concentration (%) alone by ± 10%, and (3) estimates of both DBH or BD and N concentration (%) by ± 10%.

We first estimated biomass for each transect (n = 3) in a plot by calculating the biomass of the eight or nine trees randomly sampled at 5-meter intervals along each transect, averaging their biomass, and multiplying it by stem density along the transect. We then averaged the three transects to estimate of plot level biomass. Nitrogen pools are estimated by multiplying biomass by N concentrations. Here, we focus on the sensitivity of N concentrations measured in foliar samples collected and analyzed individually from eight trees per plot then averaged at the plot level. Next, we evaluated whether results of our paired-plot tests for differences between 25 and 35 years postfire would be altered by measurement error.

Results

Differences in the estimated aboveground lodgepole pine N pool varied more as a function of tree basal diameter estimates than foliar N concentrations (Table S1). If basal diameters or foliar N concentrations were systematically 10% lower than measured, paired t-tests still revealed an

increase in lodgepole pine N pools over time ($p < 0.05$ in all cases). If all basal diameters were systematically 10% lower and all foliar N concentrations were 10% lower, paired t-tests were no longer statistically significant (paired *t*-test, $t = 1.3$, $p = 0.22$). However, each of these estimates of systematic errors are unlikely, and we conclude that small differences in individual measurements will not alter results of this study fundamentally. There is no reason to believe there would be systematic directional error in all measurements. We also note that within-plot CVs for foliar N concentration are low, ranging from 9 to 26%.

Table S2. Results of the sensitivity analyses that varied measured lodgepole pine DBH or BD (n = 25 trees per plot) and measured foliar N concentrations (*n* = 8 trees per plot) individually by ± 10% and N concentrations as well as lodgepole pine biomass by ± 10%. The original value refers to the estimated N pools presented in the main text, while the remaining columns present the low (-10%) and high (+10%) estimates of the sensitivity analysis.

	**Lodgepole pine aboveground N pool (kg ha^{-1})**			
**Plot**	**Original**	**Biomass ± 10%**	**[N] ±10%**	**[N] & Biomass ± 10%**
84blowdn	123.1	112.4 - 135.9	113.7 - 132.4	103.2 - 145.6
BiscBas	377.2	292.7 - 474.6	346.3 - 408.1	268.7 - 513.3
CascCk	83.7	72.1 - 97.2	77.5 - 89.9	66.5 - 104.1
CascMeaN	39.2	32.8 - 45.2	36.1 - 42.2	30.2 - 48.7
CascMeaS	174.5	135.5 - 219.4	160.0 – 189.0	124.2 - 237.5
CygLkNo	125.4	97.4 - 146.1	114.9 - 135.9	89.2 - 157.7
FirLopSo	140.9	109.6 - 176.9	129.1 - 152.6	100.5 - 191.6
FounEast	161.5	125.9 - 202.4	148.1 – 175.0	115.4 - 219.3
GibFalls	325.8	252.8 - 409.9	299.4 - 352.2	232.3 – 443.0
GravPit	171.0	133.2 - 214.4	156.9 - 185.1	122.2 - 232.1
LewCan	56.4	48.2 - 71.2	52.1 - 60.7	44.4 - 76.6
PitchHOB	83.2	70.1 - 97.5	77.5 - 88.8	65.1 – 104.0
RidLkHO	256.7	207.8 - 301.8	236.6 - 276.8	191.1 - 324.6
SoLewCan	220.6	171.2 – 273.0	202.8 - 238.5	157.3 - 294.6
**Mean**	167.1	133.0 - 204.7	153.6 - 180.5	122.2 - 220.9

S3.2. Uncertainty in litter and soil N pools

Approach

We calculated litter and soil N pools from biomass and N concentrations sampled in the field. Because these equations are linear, errors in mass or N concentration do not compound differently. Thus, varying biomass or N concentration individually by ± 10% yields the same results. We conducted a sensitivity analysis by varying (1) estimates of mass or N concentration (%) by ± 10%, and (2) estimates of mass and N concentration (%) by ± 10%. We also calculated CVs of mass and N concentrations for litter and N concentrations for soil samples.

Results

Variation in litter N concentration was slightly higher than foliar N concentration, with CVs ranging from 7 to 40%. Variation in litter mass was high, with within-plot CVs that ranging from 29 to 266% and were strongly related to lodgepole pine stem density (Spearman's rho = -0.84, $p < 0.001$). The most conservative estimate of litter N pools, assuming a systematic measurement error of -10% in mass and N concentration, still supported our conclusion of increasing litter N pools over time (paired *t*-test, $t = 2.6$, $p = 0.02$; Table S2).

Within-plot variation in soil N concentration ranged from 7 to 49% (CVs). If we assumed a systematic measurement error of -10% in mass and N concentration, results still supported our conclusion of no change over time in soil N pools (paired *t*-test, $t = -1.2$, $p = 0.25$; Table S3).

Table S3. Results of the sensitivity analysis that varied forest floor litter N concentrations or mass by ± 10% and N concentrations as well as mass by ± 10%. The original value refers to the estimated N pools presented in the main text, while the remaining columns present the low (-10%) and high (+10%) estimates of the sensitivity analysis.

Forest floor litter N pool (Kg N ha^{-1})			
**Plot**	**Original**	**Biomass or [N] ± 10%**	**[N] & Biomass ± 10%**
84blowdn	103.9	93 - 113.7	83.7 - 125.1
BiscBas	106.5	94.5 - 115.5	85.1 - 127.1
CascCk	34.5	30.8 - 37.7	27.7 - 41.4
CascMeaN	29.7	21.1 - 25.8	19.0 - 28.4
CascMeaS	127	118.9 - 145.3	107.0 - 159.9
CygLkNo	77.3	68.7 - 83.9	61.8 - 92.3
FirLopSo	69.2	62.2 – 76.0	56.0 - 83.6
FounEast	65.3	56.9 - 69.6	51.3 - 76.6
GibFalls	82.3	71.3 - 87.1	64.2 - 95.8
GravPit	82.7	74.8 - 91.5	67.4 - 100.6
LewCan	4.0	3.1 - 3.8	2.8 - 4.2
PitchHOB	14.9	13.3 - 16.2	12.0 - 17.9
RidLkHO	116.4	103.2 - 126.1	92.9 - 138.7
SoLewCan	39.8	34.6 - 42.3	31.1 - 46.5
**Mean**	68.1	60.5 - 73.9	54.4 - 81.3

Table S4. Results of the sensitivity analysis that varied soil N concentrations or mass by ± 10% and N concentrations as well as mass by ± 10%. The original value refers to the estimated N pools presented in the main text, while the remaining column presents the low (-10%) and high (+10%) estimates of the sensitivity analysis.

Plot	Original	Mass / [N] ± 10%	Mass & [N] ± 10%
84blowdn	871.8	784.6 - 959.0	706.2 - 1054.9
BiscBas	991.0	891.9 - 1090.1	802.7 - 1199.1
CascCk	998.0	898.2 - 1097.8	808.4 - 1207.6
CascMeaN	803.9	723.5 - 884.3	651.2 - 972.8
CascMeaS	837.0	753.3 - 920.7	678.0 - 1012.8
CygLkNo	838.6	754.7 - 922.4	679.2 - 1014.7
FirLopSo	646.9	582.2 - 711.6	524.0 - 782.7
FounEast	811.6	730.4 - 892.8	657.4 - 982
GibFalls	1083.0	974.7 - 1191.3	877.2 - 1310.4
GravPit	569.8	512.8 - 626.7	461.5 - 689.4
LewCan	1247.2	1122.5 - 1371.9	1010.2 - 1509.1
PitchHOB	1668.3	1501.5 - 1835.1	1351.3 - 2018.7
RidLkHO	1385.0	1246.5 - 1523.6	1121.9 - 1675.9
SoLewCan	1049.8	944.8 - 1154.8	850.3 - 1270.2
**Mean**	985.9	887.3 - 1084.4	798.5 - 1192.9

S3.3. Uncertainty in coarse woody debris N pools

Approach

We calculated coarse woody debris N pools from biomass sampled in the field and N concentrations obtained from the peer-reviewed literature (Fahey et al. 1985). Once dead postfire snags fall and come in contact with the soil, N concentrations in lodgepole pine woody debris increase (Fahey et al. 1985, Laiho and Prescott 1999, 2004) because of N acquisition by fungi and N fixation by bacteria (Rinne et al. 2017, Tláskal et al. 2021). Decomposing wood can remain a strong N sink for 80 – 110 years after stand initiation (Fahey et al. 1985). Loss of C via respiration during decomposition can also contribute to increasing N concentrations.

There is uncertainty in the N concentrations we used to estimate downed coarse wood N pools because we did not measure wood N concentrations in the field but relied instead on N concentrations reported in the literature (Table S5). Three studies report N concentrations in lodgepole pine downed wood by decay class, and the reported N concentrations are remarkably consistent across latitudinal and elevational gradients (Table S5). We opted for a conservative estimate of N pools in our analysis and selected the lowest reported N concentrations from the study geographically closest to our study area (Fahey 1983 in southeastern Wyoming). These concentrations are lower than the weighted mean across studies (Table S5).

Our finding that N pools in downed coarse wood increased from 25-35 years postfire is especially sensitive to variation in sound (decay classes I - II) downed wood N concentrations because no sound wood remained at 35 years postfire. To assess the sensitivity of N pools in downed coarse wood, we recalculated this pool 25 and 35 years postfire using the lowest and highest reported N concentration.

For rotten downed coarse wood, the lowest N concentration was 0.15% (mean of decay classes III – V from Busse 1994) the highest was 0.33% (decay classes III – V in Fahey 1983). For sound downed coarse wood, the lowest N concentration was 0.05% (mean of decay classes I & II in Busse 1994) and the highest was 0.13% (decay class II in Fahey 1983).

Results

Our sensitivity analysis resulted in a range of N pool sizes for 25 (70.7 – 169.0 kg N ha^{-1}) and 35 (127.7 – 281.0 kg N ha^{-1}) years postfire (Table S6). At both times postfire, the original values used in our analysis tend toward the lower end of this range, reflecting that our estimates of N pool sizes are conservative.

Table S5: Nitrogen concentrations in lodgepole pine downed wood from peer-reviewed studies. Our study sites' latitudes (44 - 45°N) and elevations (2,100 – 2,700) fall within the within the latitudinal and elevation ranges of published values. Note that Fahey (1983) reports data separately for three stands within his study region.

**Reference**	**Study area & (latitude)**	**Elevation (m above sea level)**	**N concentrations in % and (sample size, *n*) by decay class**				
			I	*II*	*III*	*IV*	*V*
Fahey 1983	Southern WY (41°N)	3,050 m	0.065 (17)	0.085 (19)	0.2 (18)	0.2 (18)	0.2 (18)
		2,900 m		0.13 (17)	0.31 (20)	0.31 (20)	0.31 (20)
		2,800 m		0.12 (20)	0.33 (15)	0.33 (15)	0.33 (15)
Wei et al. 1997	Central BC (52°–53°N)	600 m	0.08 (8)	0.08 (8)	0.1 (8)	0.22 (8)	0.22 (8)
Busse 1994	Central OR (43° 'N)	1670 m	0.04 (6)	0.06 (6)	0.08 (6)	0.18 (6)	0.18 (6)
		Weighted mean	0.064	0.103	0.26	0.26	0.26

Table S6: Results of the sensitivity analysis of downed coarse wood at 25 and 35 years postfire. The range of possible N pools (kg N ha^{-1}) was calculated using the minimum and maximum reported N concentrations by decay class for decaying lodgepole pine logs.

**Lodgepole pine downed woody debris N pool (kg ha-1)**				
	25 years postfire		35 years postfire	
**Plot**	Original	Range	Original	Range
84blowdn	69.8	47.6 - 111.6	103.9	77.9 - 171.4
BiscBas	121.6	81.4 - 193.3	120.0	90.0 - 197.9
CascCk	118.3	74.6 - 184.7	184.5	138.4 - 304.4
CascMeaN	130.4	83.4 - 204.4	274.9	206.1 - 453.5
CascMeaS	146.4	93.0 - 229.0	337.0	252.8 - 556.1
CygLkNo	125.4	78.2 - 195.1	281.1	210.8 - 463.8
FirLopSo	53.2	33.4 - 82.9	113.1	84.8 - 186.6
FounEast	64.5	43.2 - 102.6	59.5	44.7 - 98.3
GibFalls	52.1	34.7 - 82.7	70.6	53.0 - 116.6
GravPit	49.8	31.5 - 77.8	136.4	102.3 - 225.1
LewCan	136.8	94.6 - 219.7	151.3	113.5 - 249.7
PitchHOB	130.9	95.1 - 213.6	129.8	97.4 - 214.2
RidLkHO	172.1	115.3 - 273.7	228.2	171.2 - 376.6
SoLewCan	120.8	84.3 - 194.6	193.8	145.3 - 319.7
**Mean**	106.6	70.7 – 169.0	170.3	127.7 – 281.0

References

Busse, M. D. 1994. Downed Bole-Wood Decomposition in Lodgepole Pine Forests of Central Oregon. Soil Science Society of America Journal 58:221–227.

Comeau, P. G., and J. P. Kimmins. 1989. Above- and below-ground biomass and production of lodgepole pine on sites with differing soil moisture regimes. Canadian Journal of Forest Research 19:447–454.

Copenhaver, P. E., and D. B. Tinker. 2014. Stand density and age affect tree-level structural and functional characteristics of young, postfire lodgepole pine in Yellowstone National Park. Forest Ecology and Management 320:138–148.

Damgaard, C. 2020. Measurement Uncertainty in Ecological and Environmental Models. Trends in Ecology & Evolution 35:871–873.

Fahey, T. J. 1983. Nutrient dynamics of aboveground detritus in lodgepole pine (*Pinus contorta* ssp. *latifolia*) ecosystems, southeastern Wyoming. Ecological Monographs 53:51–72.

Fahey, T. J., J. B. Yavitt, J. A. Pearson, and D. H. Knight. 1985. The nitrogen cycle in lodgepole pine forests, southeastern Wyoming. Biogeochemistry 1:257–275.

Kashian, D. M., W. H. Romme, D. B. Tinker, M. G. Turner, and M. G. Ryan. 2013. Postfire changes in forest carbon storage over a 300-year chronosequence of Pinus contorta-dominated forests. Ecological Monographs 83:49–66.

Laiho, R., and C. E. Prescott. 1999. The contribution of coarse woody debris to carbon, nitrogen, and phosphorus cycles in three Rocky Mountain coniferous forests. Canadian Journal of Forest Research 29:1592–1603.

Laiho, R., and C. E. Prescott. 2004. Decay and nutrient dynamics of coarse woody debris in northern coniferous forests: a synthesis. Canadian Journal of Forest Research 34:763–777.

Litton, C. M., M. G. Ryan, and D. H. Knight. 2004. Effects of tree density and stand age on carbon allocation patterns in postfire lodgepole pine. Ecological Applications 14:460–475.

Rinne, K. T., T. Rajala, K. Peltoniemi, J. Chen, A. Smolander, and R. Mäkipää. 2017. Accumulation rates and sources of external nitrogen in decaying wood in a Norway spruce dominated forest. Functional Ecology 31:530–541.

Tláskal, V., V. Brabcová, T. Větrovský, M. Jomura, R. López-Mondéjar, L. M. Oliveira Monteiro, J. P. Saraiva, Z. R. Human, T. Cajthaml, U. Nunes da Rocha, and P. Baldrian. 2021. Complementary Roles of Wood-Inhabiting Fungi and Bacteria Facilitate Deadwood Decomposition. mSystems 6:10.1128/msystems.01078-20.

Toms, J. D., and M. L. Lesperance. 2003. Piecewise Regression: A Tool for Identifying Ecological Thresholds. Ecology 84:2034–2041.

Turner, M. G., R. E. Heumann, N. G. Kiel, J. A. Warren, and C. C. Cleveland. 2025. Reburning before recovery: Effects of short-interval fire on subalpine forest nitrogen stocks and fluxes. Ecosystems 28:1–19.

Wei, X., J. P. Kimmins, K. Peel, and O. Steen. 1997. Mass and nutrients in woody debris in harvested and wildfire-killed lodgepole pine forests in the central interior of British Columbia. Canadian Journal of Forest Research 27:148–155.

Yanai, R. D., J. J. Battles, A. D. Richardson, C. A. Blodgett, D. M. Wood, and E. B. Rastetter. 2010. Estimating uncertainty in ecosystem budget calculations. Ecosystems 13:239–248.

Yanai, R. D., T. A. Mann, S. D. Hong, G. Pu, and J. M. Zukswert. 2021. The current state of uncertainty reporting in ecosystem studies: a systematic evaluation of peer-reviewed literature. Ecosphere 12:e03535.
